# Marine algal flora of Santa Maria Island, Azores

**DOI:** 10.3897/BDJ.9.e61909

**Published:** 2021-03-25

**Authors:** Ana I Azevedo Neto, Manuela I. Parente, Eva Cacabelos, Ana Cristina Costa, Andrea Zita Botelho, Enric Ballesteros, Sandra Monteiro, Roberto Resendes, Pedro Afonso, Afonso C. L. Prestes, Rita F. Patarra, Nuno V. Álvaro, David Mila-Figueras, Raul M. A. Neto, José M. N. Azevedo, Ignacio Moreu

**Affiliations:** 1 cE3c - Centre for Ecology, Evolution and Environmental Changes/Azorean Biodiversity Group, Faculdade de Ciências e Tecnologia, Departamento de Biologia, Universidade dos Açores, 9500-321 Ponta Delgada, Açores, Portugal cE3c - Centre for Ecology, Evolution and Environmental Changes/Azorean Biodiversity Group, Faculdade de Ciências e Tecnologia, Departamento de Biologia, Universidade dos Açores 9500-321 Ponta Delgada, Açores Portugal; 2 CIBIO, Centro de Investigação em Biodiversidade e Recursos Genéticos, InBIO Laboratório Associado, Pólo dos Açores, Universidade dos Açores, Faculdade de Ciências e Tecnologia, Departamento de Biologia, 9500-321 Ponta Delgada, Açores, Portugal CIBIO, Centro de Investigação em Biodiversidade e Recursos Genéticos, InBIO Laboratório Associado, Pólo dos Açores, Universidade dos Açores, Faculdade de Ciências e Tecnologia, Departamento de Biologia 9500-321 Ponta Delgada, Açores Portugal; 3 MARE – Marine and Environmental Sciences Centre, Agência Regional para o Desenvolvimento da Investigação Tecnologia e Inovação (ARDITI), Edif. Madeira Tecnopolo, Piso 2, Caminho da Penteada, Funchal, Madeira, Portugal MARE – Marine and Environmental Sciences Centre, Agência Regional para o Desenvolvimento da Investigação Tecnologia e Inovação (ARDITI), Edif. Madeira Tecnopolo, Piso 2, Caminho da Penteada Funchal, Madeira Portugal; 4 Centre d’Estudis Avançats de Blanes-CSIC,, Acc. Cala Sant Francesc 14, 17300 Blanes, Girona, Spain Centre d’Estudis Avançats de Blanes-CSIC, Acc. Cala Sant Francesc 14, 17300 Blanes, Girona Spain; 5 Faculdade de Ciências e Tecnologia, Departamento de Biologia, Universidade dos Açores, 9500-321 Ponta Delgada, Açores, Portugal Faculdade de Ciências e Tecnologia, Departamento de Biologia, Universidade dos Açores 9500-321 Ponta Delgada, Açores Portugal; 6 IMAR/Okeanos, Departamento de Oceanografia e Pescas, Universidade dos Açores, Rua Prof. Doutor Frederico Machado, 9901-862 Horta, Açores, Portugal IMAR/Okeanos, Departamento de Oceanografia e Pescas, Universidade dos Açores, Rua Prof. Doutor Frederico Machado 9901-862 Horta, Açores Portugal; 7 Expolab - Ciência Viva Science Centre, Avenida da Ciência - Beta, n.º 8, Lagoa, São Miguel, Açores, Portugal Expolab - Ciência Viva Science Centre Avenida da Ciência - Beta, n.º 8, Lagoa, São Miguel, Açores Portugal; 8 CCMMG (Centro do Clima Meteorologia e Mudanças Globais) & IITA-A (Instituto de Investigação e Tecnologias Agrárias e do Ambiente), Universidade dos Açores, Faculdade de Ciências Agrárias, Rua Capitão João d’Ávlia – Pico da Urze, 9700-042 Angra do Heroísmo, Açores, Portugal CCMMG (Centro do Clima Meteorologia e Mudanças Globais) & IITA-A (Instituto de Investigação e Tecnologias Agrárias e do Ambiente), Universidade dos Açores, Faculdade de Ciências Agrárias Rua Capitão João d’Ávlia – Pico da Urze, 9700-042 Angra do Heroísmo, Açores Portugal; 9 N/A, Ponta Delgada, Portugal N/A Ponta Delgada Portugal

**Keywords:** Macroalgae, Azores, Santa Maria Island, new records, endemic, native, uncertain, introduced, occurrence data.

## Abstract

**Background:**

The algal flora of the Island of Santa Maria (eastern group of the Azores archipelago) has attracted interest of researchers on past occasions (Drouët 1866, Agardh 1870, Trelease 1897, Schmidt 1931, Ardré et al. 1974, Fralick and Hehre 1990, Neto et al. 1991, Morton and Britton 2000, Amen et al. 2005, Wallenstein and Neto 2006, Tittley et al. 2009, Wallenstein et al. 2009a, Wallenstein et al. 2010, Botelho et al. 2010, Torres et al. 2010, León-Cisneros et al. 2011, Martins et al. 2014, Micael et al. 2014, Rebelo et al. 2014, Ávila et al. 2015, Ávila et al. 2016, Machín-Sánchez et al. 2016, Uchman et al. 2016, Johnson et al. 2017, Parente et al. 2018). Nevertheless, the Island macroalgal flora is not well-known as published information reflects limited collections obtained in short-term visits by scientists. To overcome this, a thorough investigation, encompassing collections and presence data recording, was undertaken at both the littoral and sublittoral levels down to a depth of approximately 40 m, covering an area of approximately 64 km^2^. The resultant taxonomic records are listed in the present paper which also provides information on species ecology and occurrence around the Island, improving, thereby, the knowledge of the Azorean macroalgal flora at both local and regional scales.

**New information:**

A total of 2329 specimens (including some taxa identified only to genus level) belonging to 261 taxa of macroalgae are registered, comprising 152 Rhodophyta, 43 Chlorophyta and 66 Ochrophyta (Phaeophyceae). Of these, 174 were identified to species level (102 Rhodophyta, 29 Chlorophyta and 43 Ochrophyta), encompassing 52 new records for the Island (30 Rhodophyta, 9 Chlorophyta and 13 Ochrophyta), 2 Macaronesian endemics (*Laurencia viridis* Gil-Rodríguez & Haroun; and *Millerella tinerfensis* (Seoane-Camba) S.M.Boo & J.M.Rico), 10 introduced (the Rhodophyta *Acrothamnion preissii* (Sonder) E.M.Wollaston, *Antithamnion hubbsii* E.Y.Dawson, *Asparagopsis armata* Harvey, *Bonnemaisonia hamifera* Hariot, *Melanothamnus harveyi* (Bailey) Díaz-Tapia & Maggs, *Scinaia acuta* M.J.Wynne and *Symphyocladia marchantioides* (Harvey) Falkenberg; the Chlorophyta Codium fragile subsp. fragile (Suringar) Hariot; and the Ochrophyta *Hydroclathrus tilesii* (Endlicher) Santiañez & M.J.Wynne, and *Papenfussiella kuromo* (Yendo) Inagaki) and 18 species of uncertain status (11 Rhodophyta, 3 Chlorophyta and 4 Ochrophyta).

## Introduction

The marine algal flora of the isolated mid-Atlantic Azores archipelago is considered cosmopolitan, with species shared with Macaronesia, North Africa, the Mediterranean Sea, Atlantic Europe and America (Tittley 2003, Tittley and Neto 2006, Wallenstein et al. 2009b) and relatively rich when compared to that of other remote oceanic Islands (Neto et al. 2005, Tittley and Neto 2005, Wallenstein et al. 2009b). Amongst the Atlantic archipelagos, Azores, with 405 species, comes second in species richness after the Canary Islands, with 689 species and is followed by Madeira (396), Cabo Verde (333) and Selvagens (295 species) (Freitas et al. 2019). The latter authors, based on extensive analysis encompassing data on coastal fishes, brachyurans, polychaetes, gastropods echinoderms and macroalgae, suggested that the Azores should be a biogeographical entity on its own and proposed a re-definition of the Lusitanian biogeographical province, in which they consider four ecoregions: the South European Atlantic Shelf, the Saharan Upwelling, the Azores ecoregion and a new ecoregion they named Webbnesia, which comprises the archipelagos of Madeira, Selvagens and the Canary Islands.

It should be noted that the paper by Freitas et al. (2019) reflects data from only a few of the nine Islands, since not all data were available to them. São Miguel, with 260 algal species cited at the moment (Table 1), is the Island with the greatest amount of research dedicated to the subject. To overcome this situation and with the aim of providing a better knowledge of the archipelago’s seaweed flora, research has been conducted over the past three decades on all the Islands. Data on the Islands of Corvo and Flores, Graciosa, Pico and Terceira are already available on the recently-published papers by Neto et al. (2020a), Neto et al. (2020b), Neto et al. (2020c), Neto et al. (2020e). Table 1 summarises the current available information.

The present paper presents both physical and occurrence data and information gathered from macroalgae surveys undertaken on Santa Maria Island mainly by the Island Aquatic Research Group of the Azorean Biodiversity Centre of the University of the Azores (Link: https://ce3c.ciencias.ulisboa.pt/sub-team/island-aquatic-ecology), the BIOISLE, Biodiversity and Islands Research Group of CIBIO-Açores at the University of the Azores (Link: https://cibio.up.pt/research-groups-1/details/bioisle) and the OKEANOS Centre of the University of the Azores (Link: http://www.okeanos.uac.pt). In these surveys, particular attention was given to the small filamentous and thin sheet-like forms that are often short-lived and fast-growing and usually very difficult to identify in the wild, without the aid of a microscope and specialised literature in the laboratory.

The paper aims to provide a valuable marine biological tool for research on systematics, diversity and conservation, biological monitoring, climate change, ecology and more applied studies, such as biotechnological applications, for academics, students, government, private organisations and the general public.

## General description

### Purpose

In this paper we present taxonomic records of macroalgae for Santa Maria Island and provide general information on their occurrence and distribution. By doing this, we are addressing several biodiversity shortfalls (see Cardoso et al. 2011, Hortal et al. 2015), namely the need to catalogue the Azorean macroalgae (Linnean shortfall) and improve the current information on their local and regional geographic distribution (Wallacean shortfall), as well as on species abundance and dynamics in space (Prestonian shortfall).

## Project description

### Title

Marine algal flora of Santa Maria Island, Azores

### Personnel

Collections were conducted and occurrence data recorded during several years (1989 - 2019). Main collectors were Abel Sentíes, Afonso C. L. Prestes, Ana Cristina Costa, Ana I Neto, André Amaral, Andrea Cunha, Andrea Z. Botelho, Camille Fontaine, Catarina Santos, Cláudia Lopes, Daniela Gabriel, David Milla-Figueras, Dinis Geraldes, Edgar Rosas-Alquicira, Edward Hehre, Emanuel Xavier, Enric Ballesteros, Eunice Nogueira, Eva Cacabelos, Francisco Wallenstein, Heather Baldwin, Joana Michael, Joana Pombo, João Brum, João Ferreira, João Monteiro, José Baptista, José M. N. Azevedo, Linda Beiroldi, Luís Resendes, Marco Enoch, Manuela I. Parente, Maria Ana Dionísio, Maria Machín-Sánchez, Maria Manuel, Marlene Terra, Mutue Toyota Fujii, Nuno Vaz Álvaro, Patrícia Madeira, Paulo Torres, Pedro Monteiro, Raquel Torres, Ricardo Cordeiro, Richard Fralick, Rita F. Patarra, Ruben Couto, Rui Sousa, Sandra Monteiro, Sérgio Ávila, Tarso Costa, Tito Silva, Valeria Cassano and Viegas Pinto.

Preliminary *in situ* identifications were done by: Abel Sentíes, Ana I Neto, Andrea Z. Botelho, Daniela Gabriel, David Milla-Figueras, Edgar Rosas-Alquicira, Edward Hehre, Enric Ballesteros, Eva Cacabelos, Francisco Wallenstein, Heather Baldwin, Manuela I. Parente, Maria Machín-Sanchez, Marlene Terra, Mutue Toyota Fujii, Nuno Vaz Álvaro, Raquel Torres, Richard Fralick, Ruben Couto and Valeria Cassano.

Abel Sentíes, Ana I. Neto, Andrea Z. Botelho, David Milla-Figueras, Edgar Rosas-Alquicira, Edward Hehre, Enric Ballesteros, Eva Cacabelos, Francisco Wallenstein, Heather Baldwin, Manuela I. Parente, Maria Machín-Sanchez, Marlene Terra, Mutue Toyota Fujii, Richard Fralick and Valeria Cassano were responsible for the final species identification.

Voucher specimen management was mainly done by Afonso C.L. Prestes, Ana I. Neto, Andrea Z. Botelho, David Milla-Figueras, Eunice Nogueira, Manuela I. Parente, Natália Cabral, Rita Patarra and Roberto Resendes. Vouchers are deposited at the AZB Herbarium Ruy Telles Palhinha and the LSM - Molecular Systematics Laboratory at the Faculty of Sciences and Technology of the University of the Azores.

### Study area description

**Figure 1.**
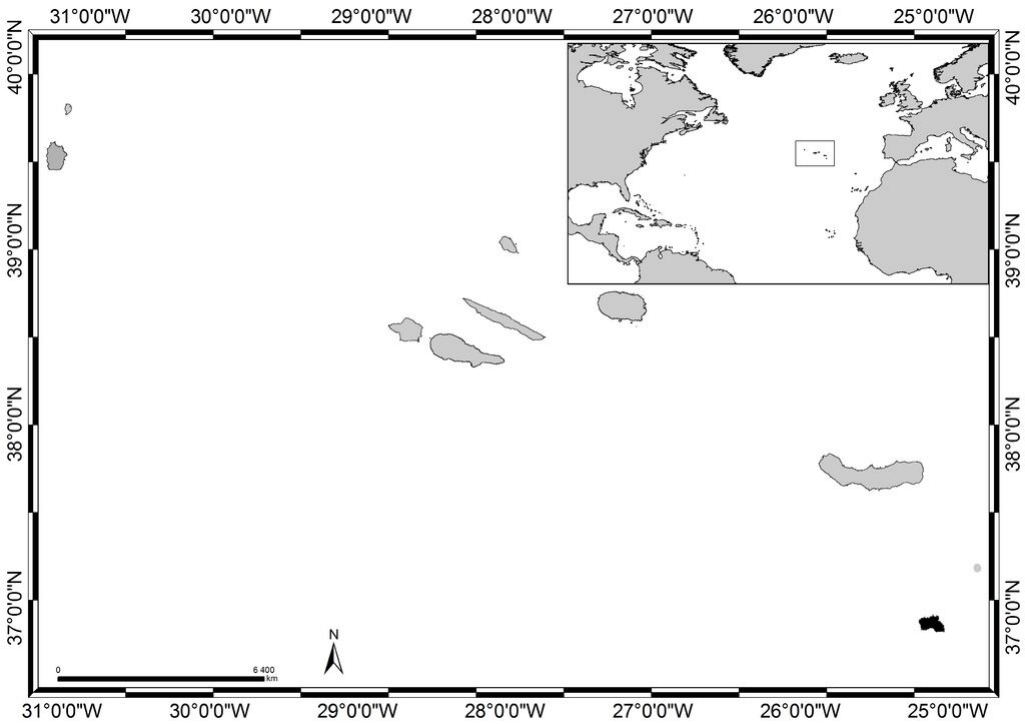
The Azores, its location in the Atlantic and Santa Maria Island highlighted in black (by Nuno V. Álvaro).

**Figure 2.**
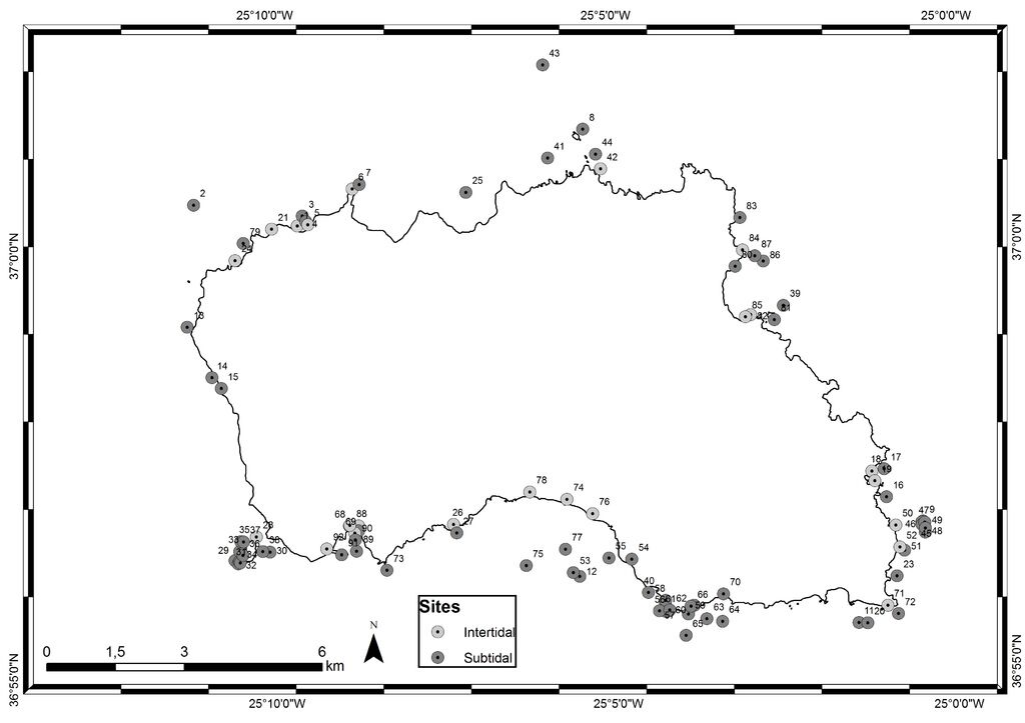
Santa Maria Island showing the sampling locations (by Nuno V. Álvaro).

Isolated in the mid-Atlantic Ocean and emerging from the Azores Plateau and located above an active triple junction between three of the world's largest tectonic plates (the North American Plate, the Eurasian Plate and the African Plate, Hildenbrand et al. 2014), the Azores archipelago (38°43′49″N, 27°19′10″W, Fig. 1) comprises nine Islands and several islets spread over 500 km in a WNW direction. The Island of Santa Maria (in black in Fig. 1), approximately 97 km², is the easternmost one of the archipelago (37°1'1''N, 25°11'6''W, Fig. 2), located approximately 430 km east of the Mid-Atlantic Ridge within the boundary that divides the Eurasian and African Plates (Hildenbrand et al. 2014). The western part of the Island is flat and has extensive wave-cut platforms reaching altitudes of 250 m above sea level. The eastern part is very irregular and has its highest point around 450 m (Neto et al. 2008c). There are no indications of recent volcanism and the last eruptions occurred during the Upper Pliocene. It is the only Island of the archipelago where marine fossiliferous deposits are known, which have been studied since the 19th century (see, for example, Amen et al. 2005, Neto et al. 2008c, Rebelo et al. 2014, Ávila et al. 2015, Ávila et al. 2016, Uchman et al. 2016).

The climate is characterised by regular rainfall, medium levels of relative humidity and persistent winds, mainly during the winter and autumn seasons (Morton et al. 1998). As in the remaining Azorean Islands, the tidal range is small (< 2 m), the coastal extension is restricted, with deep waters occurring within a few kilometres offshore and coasts are subjected to swell and surge most of the year (see Hidrográfico 1981).

The Island coastline is approximately 63 km long and the coastal morphology results from the effect of the wave action, responsible for the predominance of erosive formations and from the Island antiquity and, also, the fact that it has been frequently submerged. As a consequence, several agglomerations of marine sedimentary rocks occur (e.g. marine conglomerates, fossiliferous calcarenites and arenites) distributed through cliffs and headlands, providing a special geological value to this Island that is not present elsewhere in the archipelago (Neto et al. 2008c). The north and east coasts are characterised by discontinuous and mixed geological forms, with abrupt headlands between which lengths of large boulder and cobbles occur. At São Lourenço high cliffs give rise to narrow high-tide platforms and low headlands generally less than 10 m high, that allow the establishment of cobble beaches and marine deposition that creates the local sandy beach. The northwest coastline of the Island is characterised by the occurrence of marine deposition and agglomerations of small cobbles, while the northeast coast is sculpted by plunging cliffs. Boulders and cobbles are commonly present. The west and south coasts of the Island have predominantly steep slopes, characterised by the occurrence of plunging cliffs that vary in height, abrupt headland segments and occasional high-tide platforms covered by boulders and cobbles. Praia Formosa has a different configuration with a smooth typology that facilitates seasonal marine deposition processes that alternate between a sandy beach in summer and a cobble beach during the rest of the year (Neto et al. 2008c).

Along the coastline of the Island, the bottom is dominated by irregular rocky beds, with compact bedrock dominating over boulder and cobble ones. Only two sand basins occur, Praia Formosa (south coast) and São Lourenço on the east coast (Neto et al. 2008c). On both beaches, bedrock patches emerge from the sediment bed. This mixed substrate is common to several other places around Santa Maria, at variable depths down to 30 m (e.g. Baía do Salto de Cães and Ilhéu das Lagoinhas on the north coast, Baía do Aveiro and Baía da Maia on the east coast). Shore slope and topography show substantial variation along the shoreline. Western and northern shores are usually flatter, with depths of 30 m occurring about 500 m offshore. Eastern shores are steeper: depths of 30 m can be reached less than 200 m away from the coast. Southern shores are intermediate in this respect. The area that comprises the Praia Formosa presents a slope that is similar to that of the north side of the Island, while the one between Ponta da Malbusca and Ponta do Castelo is steeper (Neto et al. 2008c). Submerged or semi-submerged caves, arches and tunnels of small amplitude and reduced length are common. As depth increases, the slope decreases, although the bottom is still rocky and uneven (Neto et al. 2008a). The sediment floor covering the deepest areas is stable, generally composed of medium and/or coarse sand (Neto et al. 2008a). Along the coastline, natural sheltered habitats (arches and semi-submerged caves, tide pools) create favourable conditions for the growth and the occurrence of a considerable diversity and abundance of macroalgae, macroinvertebrates (Neto et al. 2008a, Neto et al. 2008b) and pelagic and benthic coastal fish (Azevedo et al. 2008).

**Figure 3.**
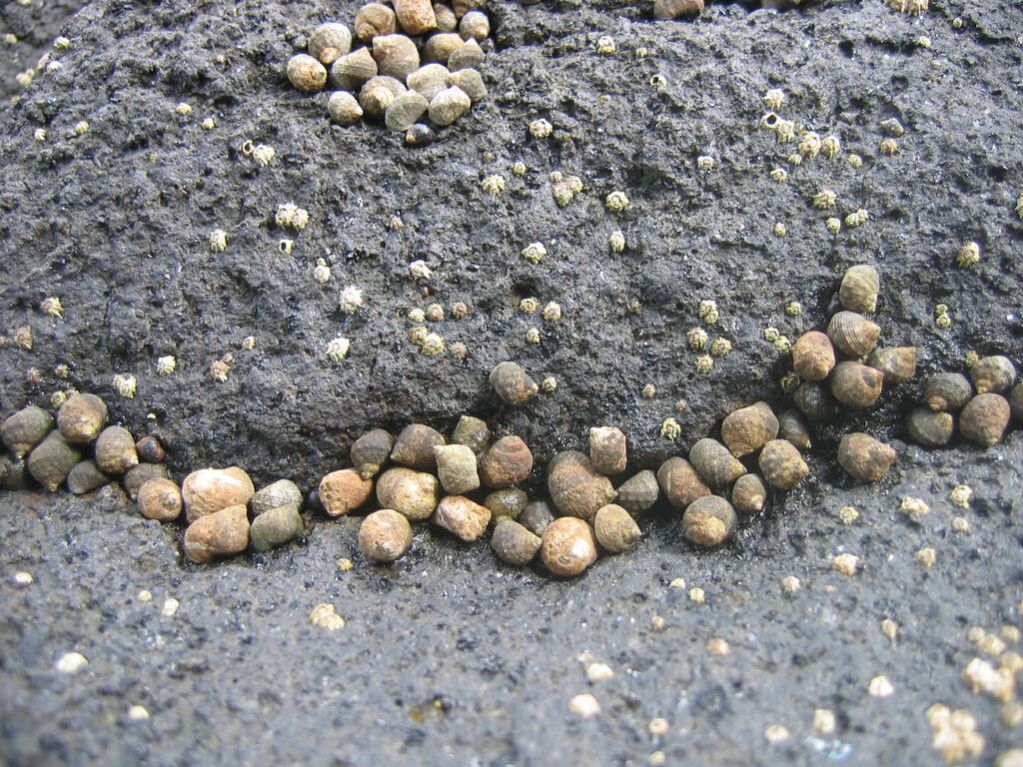
Littorinids, a characteristic species of the Azorean high intertidal level (by the Island Aquatic Ecology Subgroup of cE3c-ABG).

**Figure 4.**
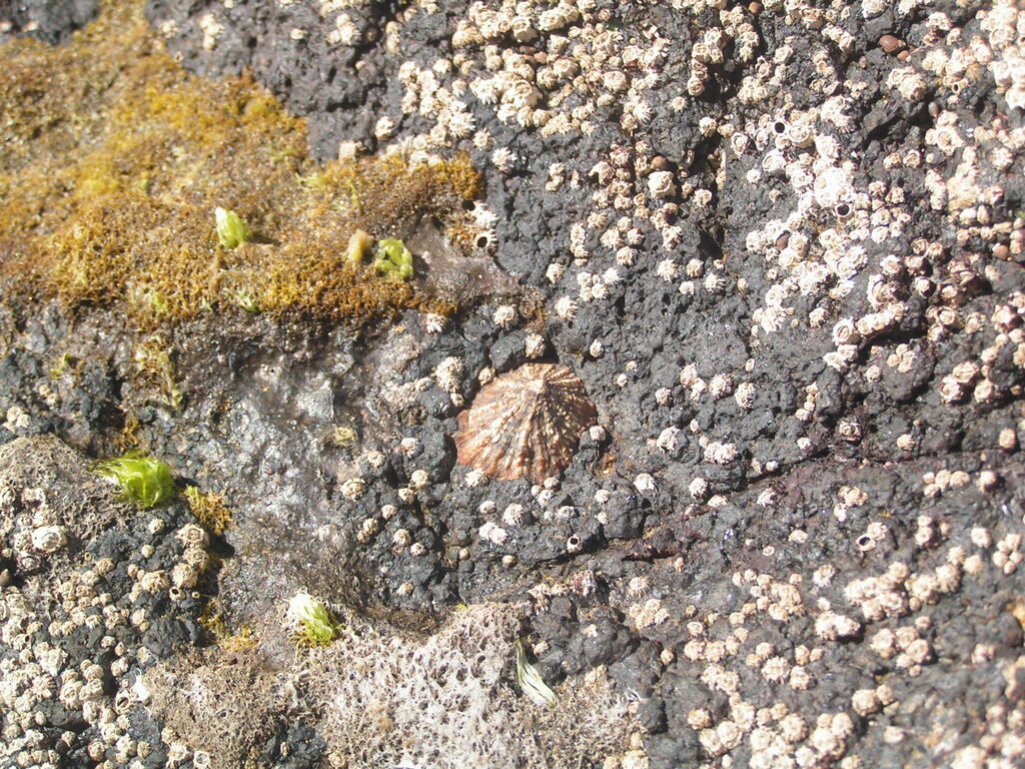
Chthamalid barnacles, algal turf and limpets on Santa Maria mid intertidal level (by the Island Aquatic Ecology Subgroup of cE3c-ABG).

**Figure 5.**
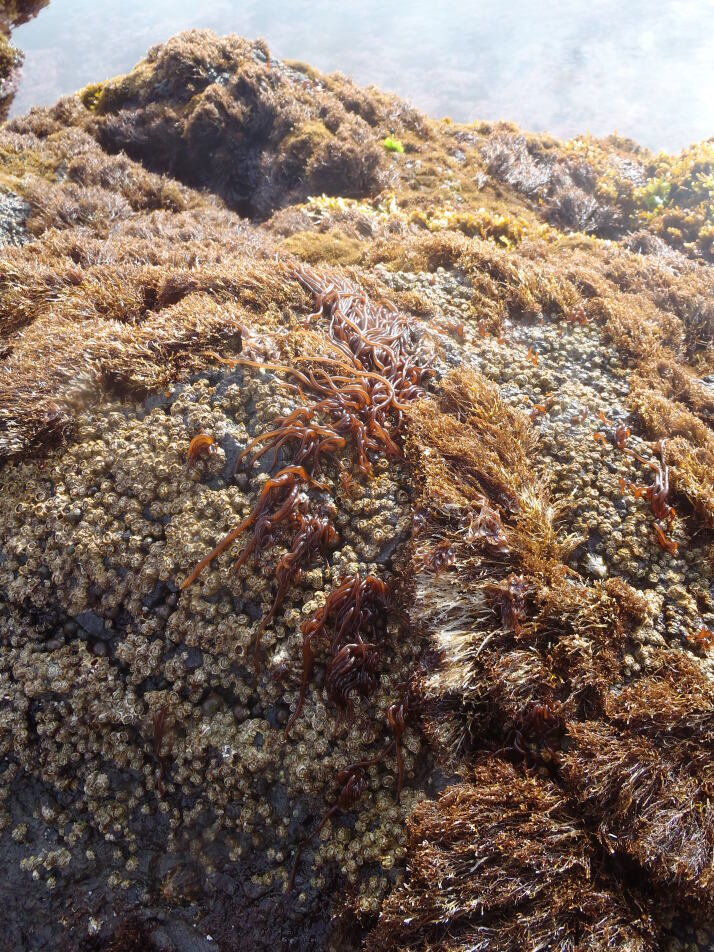
Mid-shore intertidal level, dominated by algal turf. Patches of the red algae *Nemalion elminthoides* can be seen in the image first plan (by the Island Aquatic Ecology Subgroup of cE3c-ABG).

**Figure 6.**
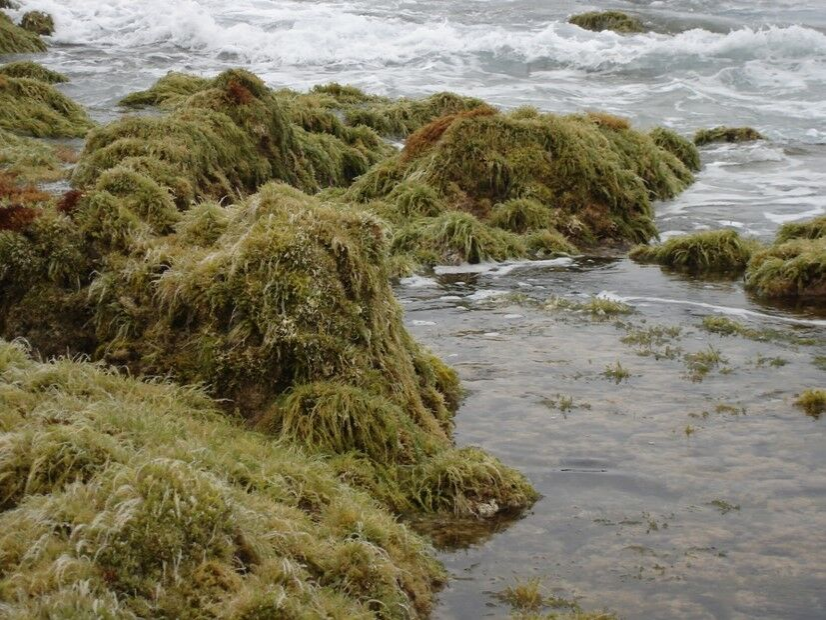
The Macaronesian endemic *Laurencia viridis* at the low-shore intertidal level (by the Island Aquatic Ecology Subgroup of cE3c-ABG).

**Figure 7.**
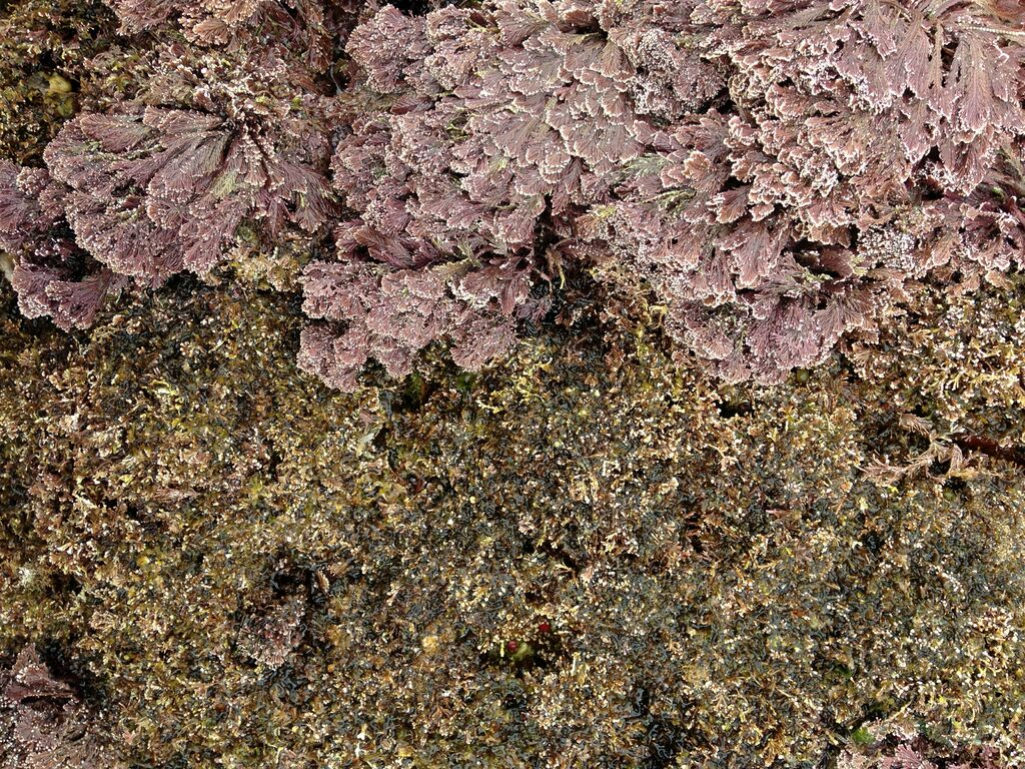
The erect calcareous frond of *Ellisolandia elongata* growing epiphytically on the algal turf at the low intertidal level (by the Island Aquatic Ecology Subgroup of cE3c-ABG).

**Figure 8.**
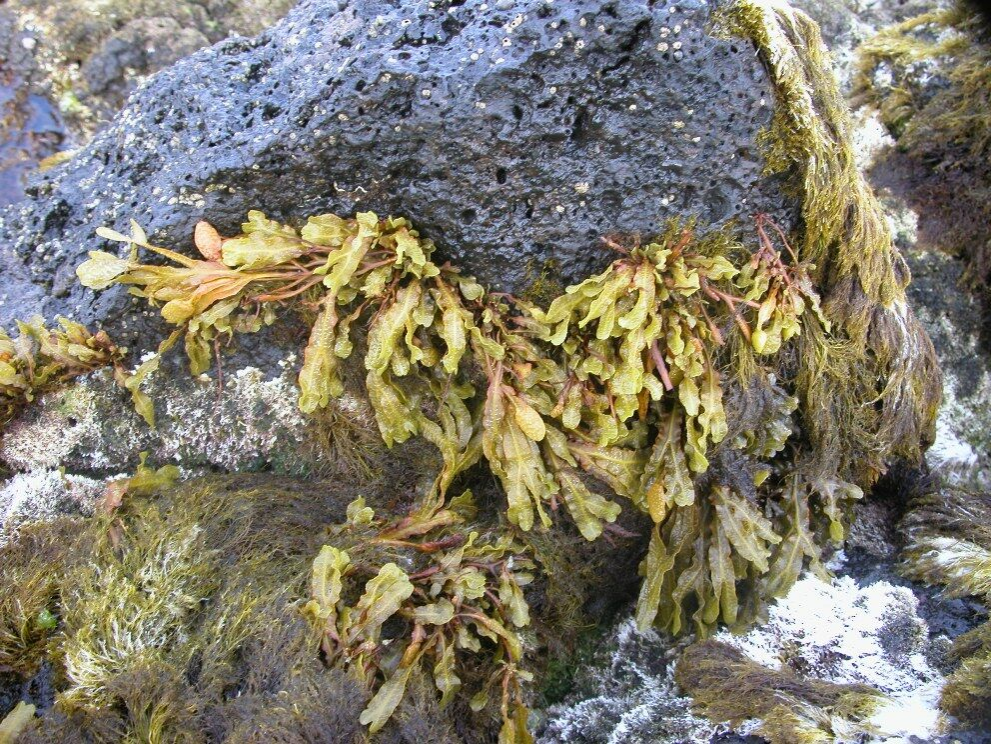
The mid-level zone on bedrock shores showing patches of the brown alga *Fucus spiralis* and the red agarophyte *Gelidium microdon* (by the Island Aquatic Ecology Subgroup of cE3c-ABG).

**Figure 9.**
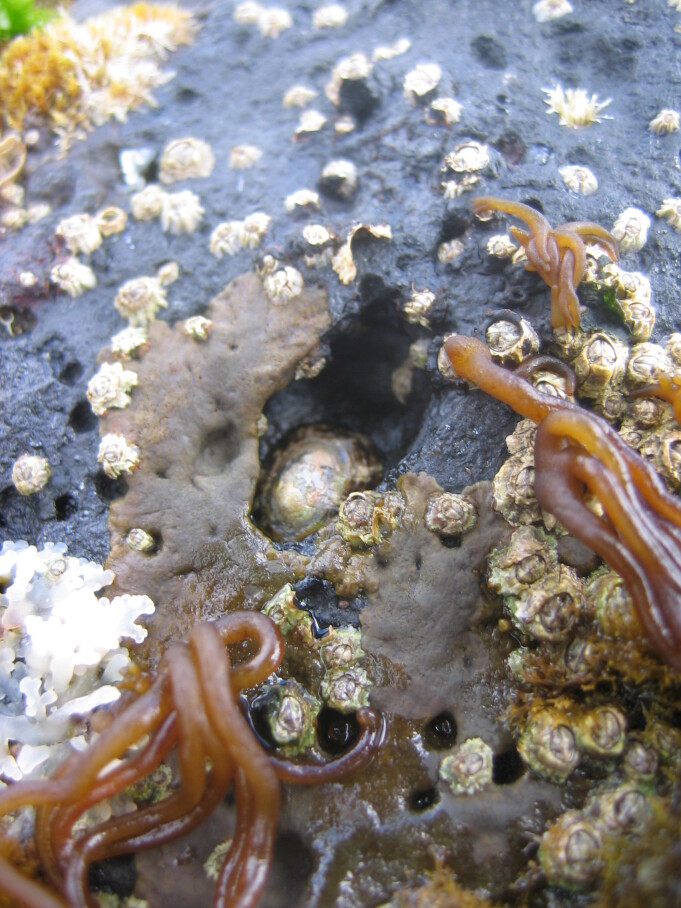
Patches of the red algae *Nemalion elminthoides* and the brown crust *Nemoderma tingitanum* at the mid-shore level of bedrock shores (by the Island Aquatic Ecology Subgroup of cE3c-ABG).

As on the other Islands of the archipelago, intertidal communities of Santa Maria Island are, in part, dominated by algal vegetation, which exhibits a distribution pattern in mosaic and/or bands, with a predominance of algal turfs, covering the rocks as a carpet (Neto et al. 2008c). This turf-growing form is a taxonomically complex mixture of small algae, recruits and juveniles of larger algae, in which the thalli intertwine and re-attach to one another and are adapted for vegetative spread using such multiple attachments to the substratum and adjacent thalli for anchorage (Wallenstein et al. 2009a). The compact mat retains water and provides a suitable habitat for admixed algae and other organisms. A very distinct horizontal pattern of species occurrence characterises the Azorean intertidal shores. In Santa Maria Island three major zones are commonly found (Neto et al. 2008c): the uppermost is dominated by littorinids (Fig. 3); the mid-level zone is characterised by chthamalid barnacles, sometimes limpets (Fig. 4) and dominated by algal turf (Fig. 5); and the lowest zone, representing the transition to the sublittoral fringe, is characterised by various species of frondose algae growing in bands (e.g. the Macaronesian endemic *Laurencia viridis*, Fig. 6), as epiphytes or forming patches amongst and over turf species (e.g. *Ellisolandia elongata* (J.Ellis & Solander) K.R.Hind & G.W.Saunders, Fig. 7). The mid-shore level zone on bedrock or boulder shores sometimes exhibits patches of the brown alga *Fucus spiralis* Linnaeus and the red agarophyte *Gelidium microdon* Kützing (Fig. 8) and/or the occasional occurrence of the red algae *Porphyra*/*Pyropia* and/or *Nemalion elminthoides* (Velley) Batters, this latter commonly growing in patches with the brown crust *Nemoderma tingitanum* Schousboe ex Bornet (Fig. 9). In spring and summer, considerable amounts of the introduced red alga *Asparagopsis armata* can be seen at the lower intertidal level.

Important features and habitats at the shore level are rock pools, occurring in different shapes and sizes and often recreating a shallow subtidal habitat which contains a rich diversity of marine life (Neto et al. 2008b). There is a gradient in the proportion of different algal groups in pools at different shore levels. Green algae dominate the upper shore while red and brown algae dominate rock pools lower on the shore. Similarly, faunal diversity in rock pools is greater at lower intertidal levels. Species diversity and richness are lower in upper shore rock-pools where climatic conditions are more stressful (Neto et al. 2008b).

**Figure 10.**
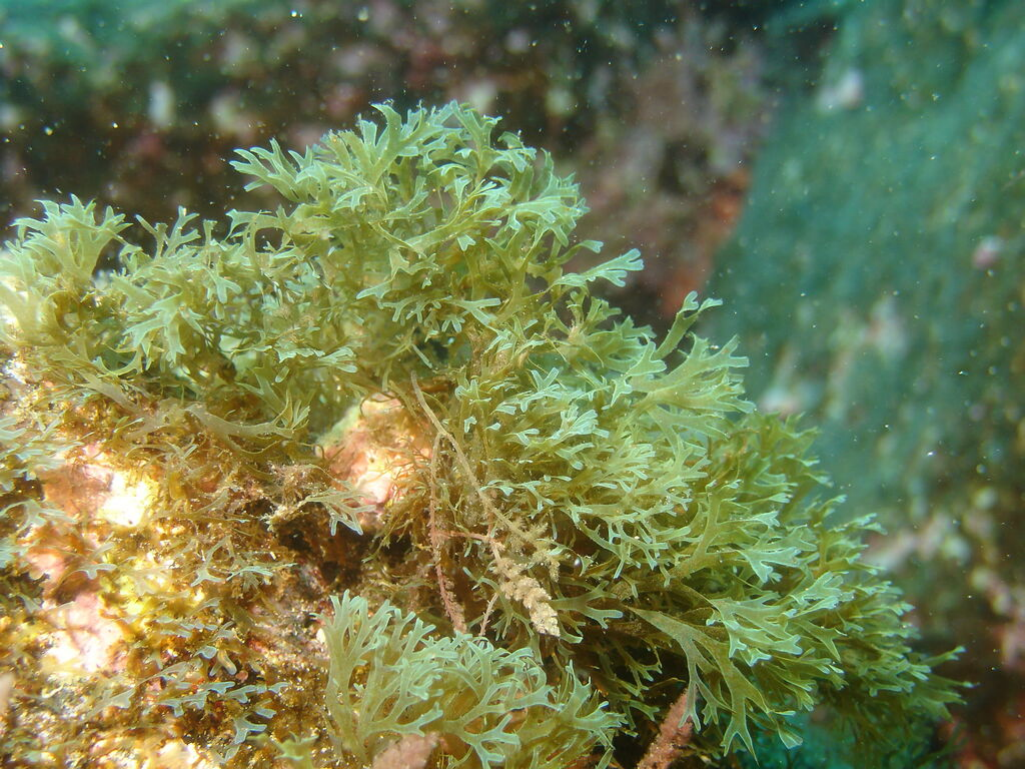
A patch of the brown alga *Dictyota* at the subtidal level (by the Island Aquatic Ecology Subgroup of cE3c-ABG).

**Figure 11.**
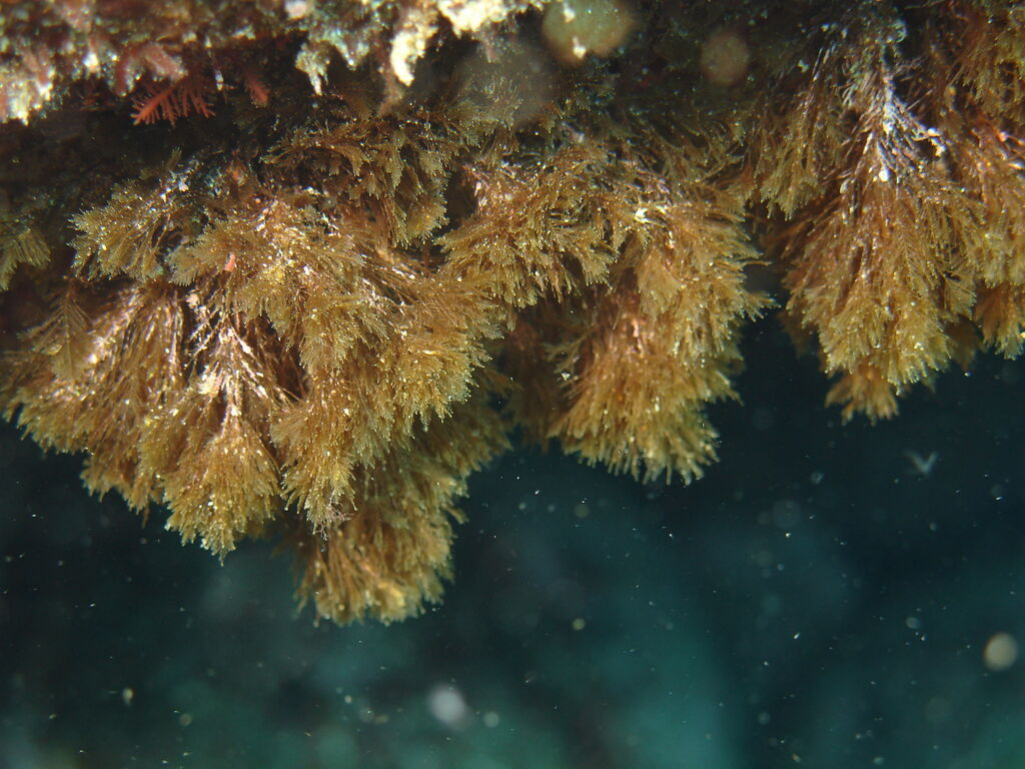
The brown alga *Halopteris filicina* at the subtidal level (by the Island Aquatic Ecology Subgroup of cE3c-ABG).

**Figure 12.**
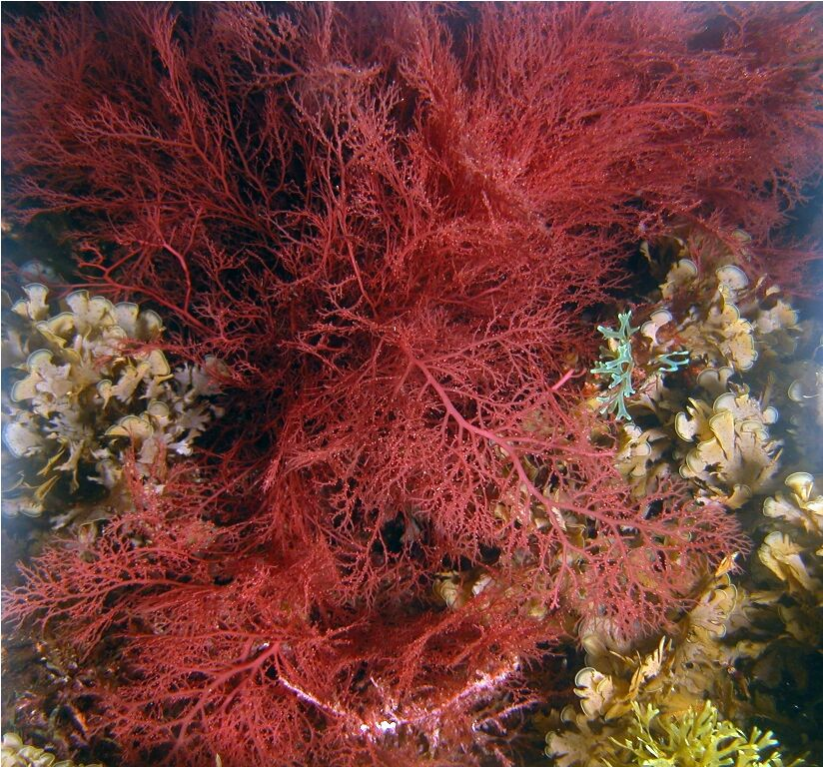
The frondose red alga *Sphaerococcus coronopifolius* growing in association with the brown algae *Zonaria tournefortii* and *Dictyota* at the deepest level sampled (by the Island Aquatic Ecology Subgroup of cE3c-ABG).

**Figure 13.**
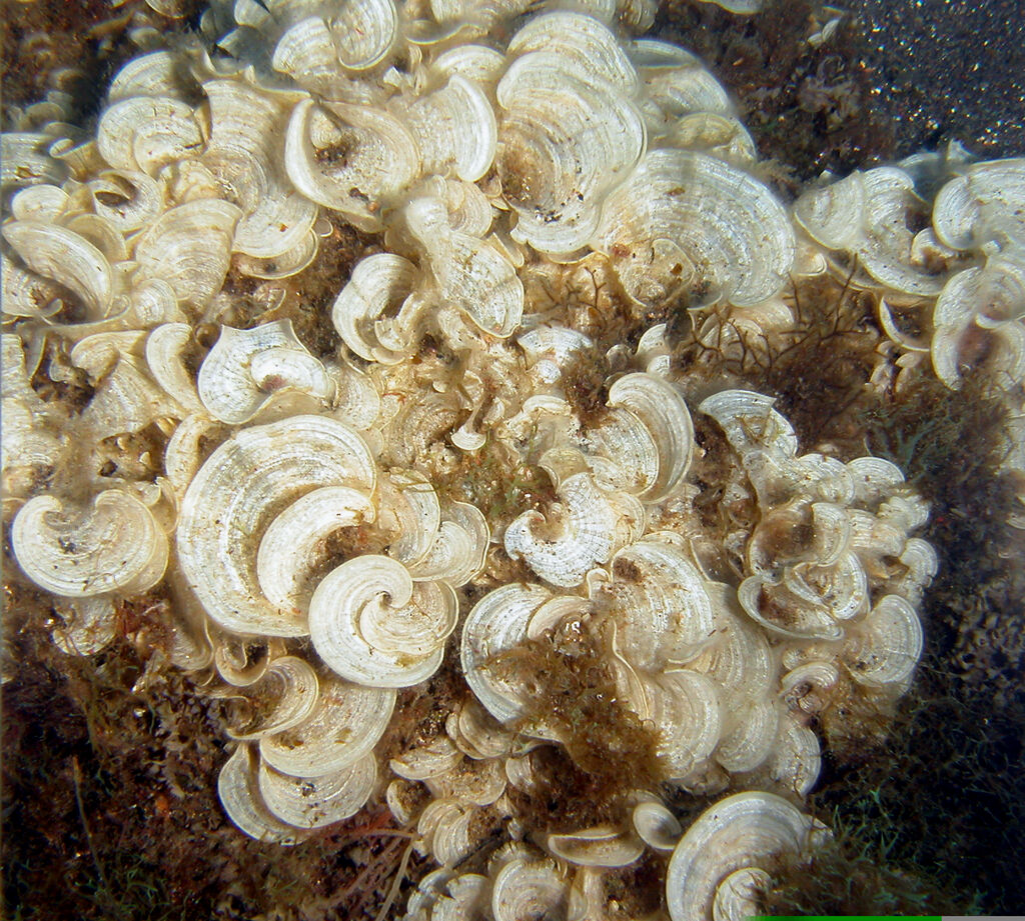
*Padina pavonica*, a locally common brown alga on the shallow bottoms of Santa Maria Island (by the Island Aquatic Ecology Subgroup of cE3c-ABG).

The rocky bottoms in the submerged zone are covered by more frondose macrophytes (Neto et al. 2008a), such as the brown algae *Dictyota* spp. (Fig. 10), *Halopteris filicina* (Grateloup) Kützing (Fig. 11), *Halopteris scoparia* (Linnaeus) Sauvageau and *Zonaria tournefortii* (J.V. Lamouroux) Montagne; and the red species *Plocamium cartilagineum* (Linnaeus) P.S. Dixon and *Sphaerococcus coronopifolius* Stackhouse (Fig. 12). The brown species *Padina pavonica* (Linnaeus) Thivy (Fig. 13) can be locally common. At this level, the edible barnacle *Megabalanus azoricus* (Pilsbry, 1916) and/or the limpet *Patella aspera* Röding, 1798 are concentrated in the first subtidal meters. Other conspicuous invertebrates are the cephalopod *Octopus vulgaris* Cuvier, 1797, the fan worm *Sabella spallanzanii* (Gmelin, 1791), the sea urchins *Sphaerechinus granularis* (Lamarck, 1816) and *Arbacia lixula* (Linnaeus, 1758) and the sea stars *Marthasterias glacialis* (Linnaeus, 1758) and *Ophidiaster ophidianus* (Lamarck, 1816) (Neto et al. 2008a). Frequent fish species at this level are the blue wrasse *Symphodus caeruleus* (Azevedo, 1999) or the ornate wrasse *Thalassoma pavo* (Linnaeus, 1758) in shallow rocky areas and the morays, *Muraena helena* Linnaeus, 1758 or the forkbeards *Phycis phycis* (Linnaeus, 1766), mainly hidden in crevices during the day. The parrotfish *Sparisoma cretense* (Linnaeus, 1758), the salemas *Sarpa salpa* (Linnaeus, 1758) and the white sea bream *Diplodus sargus* (Linnaeus, 1758) roam amongst rocky reefs (Azevedo et al. 2008).

### Design description

The sampling referred to in this paper was performed across littoral and sublittoral levels down to approximately 40 m on the Island of Santa Maria. Each sampling location was visited several times and, on each occasion, a careful and extensive survey was undertaken to provide a good coverage of the area. Both physical collections and presence recording were made by walking over the intertidal shores during low tides or by SCUBA diving. The specimens collected were taken to the laboratory for identification and preservation and the resulting vouchers were deposited at the AZB Herbarium Ruy Telles Palhinha and the LSM - Molecular Systematics Laboratory at the Faculty of Sciences and Technology of the University of the Azores.

### Funding

This study was mainly financed by the following projects/scientific expeditions:

- Projects:
  - CAJFQ – “Characterization of the algal component of quaternary fossil deposits”, integrated in the project “Macaronésia 2000”, funded by the Autonomous Organism of Museums and Centers of Tenerife, Canary Islands (1999-2004);
  - PARQMAR – “Characterization, Planning and Management of Marine Protected Areas in Macaronesia - The cases of the Eco-Marine Park of Funchal (Madeira), Gran Canaria and Tenerife (Canary Islands) and Santa Maria (Azores)”, funded by INTERREG III B 2000 Community Initiative Program - 2006, Azores-Madeira-Canary Islands. 03/ MAC/ 4.2/ M9 (2004-2006);
  - RRASMA – “Removal of abandoned fishing nets off the island of Santa Maria”, funded by the Regional Government of the Azores, Environment Delegation of Santa Maria Island (2005-2007);
  - RCGO - “Coastal Waste of the Eastern Group (São Miguel and Santa Maria Islands; Formigas Islets): inventory, catalog, raise awareness”, funded by QUERCUS (2006);
  - CAMAG/ORI – “Characterization of coastal water bodies on the islands of Santa Maria and São Miguel”, funded by the Regional Government of the Azores, Regional Secretariat for the Environment and the Sea, Regional Directorate for Planning and Water Resources (2008-2012);
  - LAUMACAT - “Diversity and phylogenetic relationships on the benthic marine algae with pharmacological potential: the *Laurencia* complex (Rhodophyta) in Macaronesian archipelagos, tropical and subtropical Atlantic”, funded by the Ministerio de Ciencia e Innovación, Dirección General de Investigación y Gestión del Plan Nacional de R+D+i, Subdirección General de Proyectos de Investigación, Gobierno de España (2010 to 2013) and by the São Paulo State Research Support Foundation (FAPESP), Brazil, Proc. 2014 / 00012-1 (2013 a 2016);
  - ASMAS - Açores: Stop-over for Marine Alien Species?” Government of the Azores - Regional Secretariat for the Sea, Science and Technology (M2.1.2/I/032/2011). 2012 – 2016;
  - PIMA – “Elaboration of the implementation program of the Marine Strategy Framework Directive - Marine Invasion Program in the Azores” (3/DRAM /2015). Government of the Azores - Regional Secretariat for the Sea, Science and Technology, Regional Directorate for Sea Affairs (GRA /SRMCT-DRAM), 2015;
  - BALA – “Elaboration of the implementation program of the marine strategy framework directive - biodiversity of the coastal environments of the Azores” (2 /DRAM /2015). Government of the Azores - Regional Secretariat for the Sea, Science and Technology, Regional Directorate for Sea Affairs (GRA /SRMCT-DRAM), 2015;
  - “ACORES-01-0145-FEDER-000072 - AZORES BIOPORTAL – PORBIOTA. Operational Programme Azores 2020 (85% ERDF and 15% regional funds);

- Scientific Expeditions and campaigns:
  - “SANTA MARIA E FORMIGAS/90”, organised by the Biology Department of the University of the Azores, Santa Maria Island, Azores, June 1990;
  - “Fossil deposits of Prainha and Lagoinhas” under the project CAJFQ- Macaronésia 2001
  - “Santa Maria 2002”, under the workshop "Marine Fossils of the Azores: Perspectives for the future", 2002;
  - “Santa Maria 2005”, under the project PARQMAR, 2005;
  - “Santa Maria Island (Azores) 2009”, organised by the Biology Department of the University of the Azores 2009;
  - “Laurencia/2011”, under the project LAUMACAT, 2011;
  - “Waitt Foundation”, under the projects BALA and PIMA, 2016;
  - “BALA/PIMA”, under the projects BALA and PIMA, 2018;
  - “PORBIOTA/2019” under the project ACORES-01-0145-FEDER-000072 - AZORES BIOPORTAL – PORBIOTA, 2019;
- Other funds:
  - Portuguese National Funds, through FCT – Fundação para a Ciência e a Tecnologia, within the projects UID/BIA/00329/2013, 2015-2019, UID/BIA/00329/2020-2023 and UID/BIA/50027/2019, UID/BIA/50027/2013-2020 and POCI-01-0145-FEDER-006821;
  - ERDF funds through the Operational Programme for Competitiveness Factors – COMPETE;
  - Portuguese Regional Funds, through DRCT - Regional Directorate for Science and Technology, within several projects, 2019 and 2020 and SRMCT /DRAM - Regional Secretariat for the Sea, Science and Technology, Regional Directorate for Sea Affairs;
  - CIRN/DB/UAc (Research Centre for Natural Resources, Universidade dos Açores, Departamento de Biologia);
  - CIIMAR (Interdisciplinary Centre of Marine and Environmental Research, Porto, Portugal).

## Sampling methods

### Study extent

The present paper includes sampling performed on a relatively large area, of approximately 64 km^2^, covering littoral and sublittoral levels down to approximately 40 m around the Island (Table 2, Fig. 2).

### Sampling description

**Figure 14.**
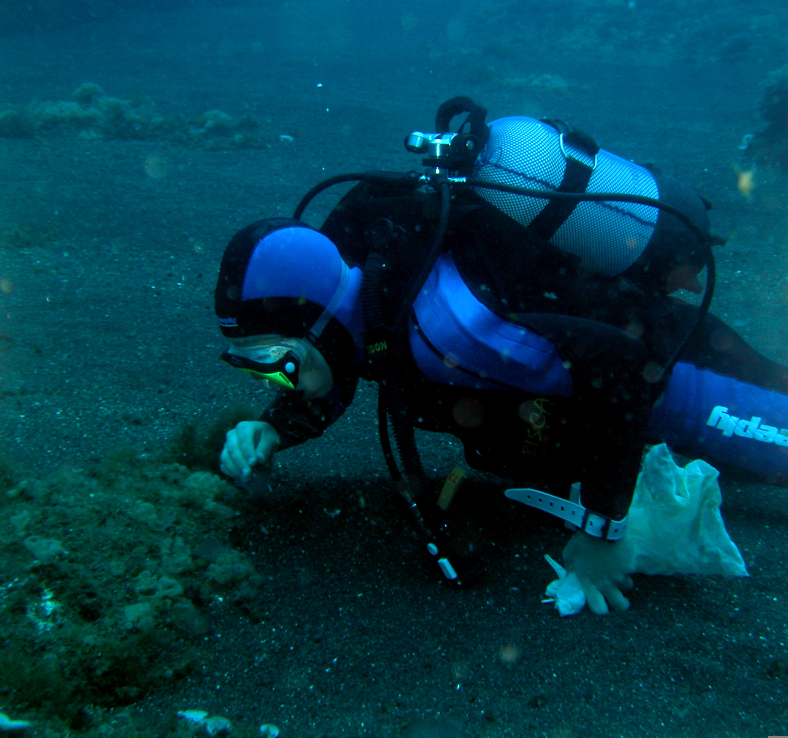
Collecting macroalgae at the subtidal of Santa Maria Island (by the Island Aquatic Ecology Subgroup of cE3c-ABG).

**Figure 15.**
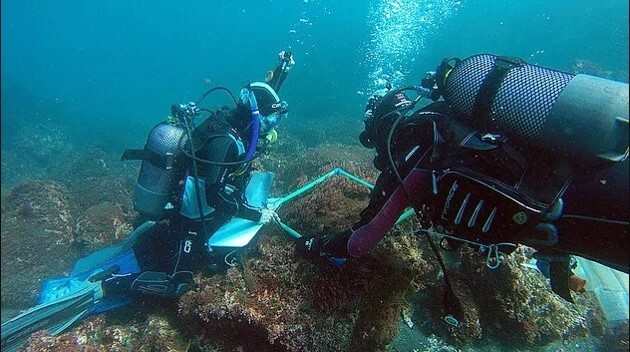
Quantitative recording of the presence and coverage of macroalgal species from subtidal rocky habitat (by the Island Aquatic Ecology Subgroup of cE3c-ABG).

Sampling involved specimen collecting and species presence recording. At each location, samples were obtained by scraping and/or manually collecting one or two specimens of all different species found into labelled bags (Fig. 14). Species recording data were gathered by registering all species present in the sampled locations (Fig. 15). Intertidal collections were made during low tide by walking over the shores. Subtidal collections were made by SCUBA diving around the area.

### Quality control

Each sampled taxon was identified by trained taxonomists and involved morphological and anatomical observations of whole specimens by eye and/or of histological preparations under microscopes to determine the main diagnostic features of each species as described in literature.

### Step description

At the laboratory, standard procedures were followed in specimens sorting and macroalgae identification. A combination of morphological and anatomical characters and reproductive structures was used for species identification. For small and simple thalli, this required the observation of the entire thallus with the naked eye and/or using dissecting and compound microscopes. For larger and more complex algae, investigation of the thallus anatomy required histological preparations (longitudinal and transverse sections) or squashed preparations of mucilaginous thalli, sometimes after staining, to observe vegetative and reproductive structures and other diagnostic features.

The Azorean algal flora has components from several geographical regions which implies difficulties in species identification. Floras and keys for the North Atlantic, Tropical Atlantic and Western Mediterranean were used (e.g. Schmidt 1931, Taylor 1967, Taylor 1978, Levring 1974, Dixon and Irvine 1977, Lawson and John 1982, Irvine 1983, Gayral and Cosson 1986, Fletcher 1987, Afonso-Carrillo and Sansón 1989, Burrows 1991, Boudouresque et al. 1992, Cabioc'h et al. 1992, Maggs and Hommersand 1993, Irvine and Chamberlain 1994, Brodie et al. 2007, Lloréns et al. 2012, Rodríguez-Prieto et al. 2013). For more critical and taxonomically difficult taxa, specimens were taken to the Natural History Museum (London) for comparison with collections there.

A reference collection was made for all collected specimens by assigning them a herbarium code number and depositing them at the AZB Herbarium Ruy Telles Palhinha and the LSM - Molecular Systematics Laboratory, University of Azores. Depending on the species and on planned further research, different types of collections were made, namely (i) wet collections using 5% buffered formaldehyde seawater and then replacing it by the fixing agent Kew (Bridsen and Forman 1999); (ii) dried collections, either by pressing the algae (most species) as described by Gayral and Cosson (1986) or by letting them air dry (calcareous species); and (iii) silica gel collections for molecular study.

Nomenclatural and taxonomic status used here follow *Algaebase* (Guiry and Guiry 2020). The database was organised on FileMaker Pro.

## Geographic coverage

### Description

**Santa Maria Island Description**: Azores, Portugal (approximately 37°1'19''N, -25°11'24''W);

### Coordinates

36.918 and 37.022 Latitude; -25.190 and -25.009 Longitude.

## Taxonomic coverage

### Description

All macroalgae were identified to genus or species level. In total, 261 taxa were identified belonging to 28 orders and 60 families, in the phyla Rhodophyta (14 orders and 34 families), Chlorophyta (5 orders and 9 families) and Ochrophyta (9 orders and 17 families).

## Temporal coverage

### Notes

The sampling was performed on several occasions in the period between 1989 and 2019.

## Collection data

### Collection name

AZB | Marine macroalgae collection of Santa Maria Island (Azores)-Expedition Santa Maria and Formigas/90; AZB | Marine macroalgae collection of Santa Maria Island (Azores)-Project LAUMACAT; AZB | Marine macroalgae collection of Santa Maria Island (Azores)-Project PARQMAR; AZB | Marine macroalgae collection of Santa Maria Island (Azores)-Occasional sampling; LSM | Marine macroalgae collection of Santa Maria Island (Azores)-Department of Biology Expedition 2009; LSM | Marine macroalgae collection of Santa Maria Island (Azores)-Project ASMAS; LSM | Marine macroalgae collection of Santa Maria Island (Azores)-Occasional sampling; Marine macroalgae occurrence of Santa Maria Island (Azores)-Campaign CAMAG-ORI-SMA/2008; Marine macroalgae occurrence of Santa Maria Island (Azores)-Project LAUMACAT; Marine macroalgae occurrence of Santa Maria Island (Azores)-Occasional sampling; Marine macroalgae occurrence of Santa Maria Island (Azores)-Campaign Waitt Foundation - BALA /PIMA /2016; Marine macroalgae occurrence of Santa Maria Island (Azores)-PIMA / 2016; Marine macroalgae occurrence of Santa Maria Island (Azores)-PIMA / 2017; Marine macroalgae occurrence of Santa Maria Island (Azores)-Campaign BALA /PIMA /2018; Marine macroalgae occurrence of Santa Maria Island (Azores)-Campaign Porbiota/ 2019.

### Collection identifier

81c64926-4d75-429d-b21f-f7cd93e30504; 100ab0f2-7f8b-4eb6-a5f5-6257d32003a5; af962795-47c6-4219-a295-6687a94afeda; 08883948-f896-495f-ab3d-9fe49f23b76c; 865b91e9-1ec6-4bb8-a941-aba2b586071a; 4efe744e-1e38-431c-b112-7fb9f9bf279a; 77a28947-47d8-420f-b40d-f49e87556090; 6606098f-5fbb-4731-9cfa-b7c8e78c3638; bae7fc8f-6333-43d4-887b-3e65617df133; 579bc266-7779-49ea-a775-f44abc2bdad3; 30ed893c-b66d-4c85-8848-10f144a6f957; 852eacdf-977e-44dd-9a52-172a5082a6dd; b74c3414-e277-4789-8806-27a9abf0f7ee; 22941d45-0678-49fb-bdfe-8b0052ceb298; 93e46396-33b2-4dff-b3d1-acff7e76753c.

### Parent collection identifier

AZB Herbarium Ruy Telles Palhinha, Faculty of Sciences and Technology of the University of the Azores; AZB Herbarium Ruy Telles Palhinha, Faculty of Sciences and Technology of the University of the Azores; AZB Herbarium Ruy Telles Palhinha, Faculty of Sciences and Technology of the University of the Azores; AZB Herbarium Ruy Telles Palhinha, Faculty of Sciences and Technology of the University of the Azores; LSM - Molecular Systematics Laboratory, Faculty of Sciences and Technology of the University of the Azores; LSM - Molecular Systematics Laboratory, Faculty of Sciences and Technology of the University of the Azores; Not applicable; Not applicable; Not applicable; Not applicable; Not applicable; Not applicable; Not applicable; Not applicable; Not applicable.

### Specimen preservation method

Air dry, Dried and pressed; Wet (Formalin; fixing agent Kew), Silica gel.

## Usage licence

### Usage licence

Creative Commons Public Domain Waiver (CC-Zero)

## Data resources

### Data package title

Marine algal flora of Santa Maria Island, Azores

### Resource link

https://www.gbif.org/dataset/38c70a82-c6e3-4ef4-89f4-a37455c6f73a

### Alternative identifiers

http://ipt.gbif.pt/ipt/resource?r=santa_maria_macroalgal_flora

### Number of data sets

1

### Data set 1.

#### Data set name

Marine algal flora of Santa Maria Island, Azores

#### Data format

Darwin Core Archive

#### Number of columns

50

#### Download URL

http://ipt.gbif.pt/ipt/resource?r=santa_maria_macroalgal_flora&v=1.3

#### Data format version

1.3

#### Description

This data paper presents physical and occurrence data from macroalgal surveys undertaken on Santa Maria Island between 1989 and 2019 (Neto et al. 2020d). The dataset submitted to GBIF is structured as a sample event dataset, with two tables: event (as core) and occurrences. The data in this sampling event resource have been published as a Darwin Core Archive (DwCA), which is a standardised format for sharing biodiversity data as a set of one or more data tables. The core data table contains 139 records (eventID). The extension data table has 2329 occurrences. An extension record supplies extra information about a core record. The number of records in each extension data table is illustrated in the IPT link. This IPT archives the data and thus serves as the data repository. The data and resource metadata are available for downloading in the downloads section.

**Data set 1.** 

Column label	Column description
eventID	Identifier of the event, unique for the dataset
country	Country of the sampling site
countryCode	Code of the country where the event occurred
stateProvince	Name of the region
island	Name of the island
municipality	Name of the municipality
locality	Name of the locality
locationID	Identifier of the location
decimalLatitude	The geographic latitude of the sampling site
decimalLongitude	The geographic longitud of the sampling site
geodeticDatum	The spatial reference system upon which the geographic coordinates are based
coordinateUncertaintyInMetres	The horizontal distance (in metres) from the given decimalLatitude and decimalLongitude describing the smallest circle containing the whole of the Location
eventDate	Time interval when the event occurred
year	The year of the event
samplingProtocol	Sampling method used during an event
locationRemarks	Zonation level
minimumDepthInMetres	The minimum depth in metres where the specimen was found
maximumDepthInMetres	The maximum depth in metres where the specimen was found
eventRemarks	Notes about the event
occurrenceID	Identifier of the record, coded as a global unique identifier
institutionID	The identifier for the institution having custody of the object or information referred to in the record
institutionCode	The acronym of the institution having custody of the object or information referred to in the record
collectionID	An identifier of the collection to which the record belongs
collectionCode	The name of the collection from which the record was derived
datasetName	The name identifying the dataset from which the record was derived
kingdom	Kingdom name
phylum	Phylum name
class	Class name
order	Order name
family	Family name
genus	Genus name
specificEpithet	The name of the first or species epithet of the scientificName
infraspecificEpithet	The name of the lowest or terminal infraspecific epithet of the scientificName, excluding any rank designation
acceptedNameUsage	The specimen accepted name, with authorship
previousIdentifications	Previous name of the specimen, with authorship
scientificName	The name without authorship applied on the first identification of the specimen
scientificNameAuthorship	The authorship information for the scientificName formatted according to the conventions of the applicable nomenclaturalCode
taxonRank	The taxonomic rank of the most specific name in the scientificName
basisOfRecord	The specific nature of the data record
habitat	Description of the habitat where the specimen was found
organismQuantityType	The type of quantification system used to quantity the organisms
organismQuantity	Percentage of the organism coverage
recordedBy	Person(s) responsible for sampling
catalogNumber	Identifying code for a unique sample lot in a biological collection
identifiedBy	Person(s) responsible for taxa identification
type	The nature of the resource
preparations	The preservation method used for the specimen
establishmentMeans	The establishment status of the organism in the study region
occurrenceRemarks	New record status assignment
licence	Reference to the licence under which the record is published

## Additional information

This paper is based on 2329 specimens of macroalgae recorded from Santa Maria Island in 261 taxa, comprising 174 confirmed species (Table 3) and 86 taxa identified only to genus level. The confirmed species (Table 4) include 102 Rhodophyta, 29 Chlorophyta and 43 Ochrophyta (Phaeophyceae). Of these, 52 species are newly recorded to the Island (30 Rhodophyta, 9 Chlorophyta and 13 Ochrophyta). Most species are native, including the two Macaronesian endemics (*Laurencia viridis* and *Millerella tinerfensis*). Eighteen have an uncertain status (11 Rhodophyta, 3 Chlorophyta and 4 Ochrophyta) and ten species represent introductions to the algal flora (the Rhodophyta *Acrothamnion preissii*, *Antithamnion hubbsii*, *Asparagopsis armata*, *Bonnemaisonia hamifera*, *Melanothamnus harveyi*, *Scinaia acuta* and *Symphyocladia marchantioides*; the Chlorophyta Codium fragile subsp. fragile; and the Ochrophyta *Hydroclathrus tilesii* and *Papenfussiella kuromo*).

Many species were only sporadically recorded, but 12 were commonly found around the Island and occurred quite abundantly in some locations, namely: the Rhodophyta *Asparagopsis taxiformis* (Delile) Trevisan, *Laurencia viridis*, and *Pterocladiella capillacea* (S.G. Gmelin) Santelices & Hommersand; the Chlorophyta *Cladophora prolifera* (Roth) Kützing, *Codium adhaerens* C. Agardh and *Ulva rigida* C. Agardh; and the Ochrophyta *Cladostephus spongiosus* (Hudson) C. Agardh, *Colpomenia sinuosa* (Mertens ex Roth) Derbès & Solier, *Halopteris scoparia*, *Lobophora variegata* (J.V. Lamouroux) Womersley ex E. C. Oliveira, *Padina pavonica* and *Zonaria tournefortii*.

A mismatch regarding the GBIF backbone taxonomy of some of the macroalgae species names was identified as detailed in Suppl. material 1.

## Supplementary Material

D38A3657-FA82-5DE0-B37A-6E0ACA8F3BC3 10.3897/BDJ.9.e61909.suppl1 Supplementary material 1 DP-SMA-id_15162_normalized.csv Data type Macroalgae taxonomic mismatching Brief description GBIF does not have the more actualised nomenclature for some of the macroalgae species names. Therefore, the matching tools of its platform were applied to the species list, as required by Pensoft's data auditor, to identify the problematic taxonomic situations. The resulting file (DP-SMA-id_15162_normalized.csv) is included here, since the names will not be immediately updated in the GBIF Taxonomic Backbone. A request was already sent to GBIF helpdesk to solve this situation. File: oo_477086.csv https://binary.pensoft.net/file/477086 Ana I Neto

## Figures and Tables

**Table 1.** Number of macroalgal species on the Azorean Islands: Santa Maria, São Miguel, São Jorge and Faial (authors' unpublished data); Terceira (Neto et al. 2020a); Graciosa (Neto et al. 2020c); Pico (Neto et al. 2020b); Flores and Corvo (Neto et al. 2020e).

Phyllum	Santa Maria	São Miguel	Terceira	Graciosa	São Jorge	Pico	Faial	Flores	Corvo
Rhodophyta	68	168	73	126	35	142	59	120	30
Chlorophyta	20	39	24	31	17	41	16	35	9
Ochrophyta	28	53	16	38	10	42	8	41	17
Total	116	260	113	195	62	225	83	196	56

**Table 2.** Information and location of the sampling sites on Santa Maria Island.

Location N0	Location ID	Municipality	Locality	Latitude	Longitude	Littoral zone
1	SMA_VP_Aapem	Vila do Porto	Anjos | Atrás do porto | Entre-marés	37.004998	-25.159629	Intertidal
2	SMA_VP_aaprs	Vila do Porto	Atrás do aeroporto | Ponta do Rochedo | Subtidal	36.985484	-25.187049	Subtidal
3	SMA_VP_aas1	Vila do Porto	Atrás do aeroporto | Subtidal 1	36.975484	-25.181233	Subtidal
4	SMA_VP_aas2	Vila do Porto	Atrás do aeroporto | Subtidal 2	36.973329	-25.179014	Subtidal
5	SMA_VP_Abjls	Vila do Porto	Anjos | Banco João Lopes | Subtidal	37.00946	-25.18495	Subtidal
6	SMA_VP_Abs	Vila do Porto	Aveiro | Baía | Subtidal	36.949447	-25.016892	Subtidal
7	SMA_VP_Afpis1	Vila do Porto	Anjos | Frente à Piscina | Subtidal 1	37.006907	-25.158392	Subtidal
8	SMA_VP_Afpis2	Vila do Porto	Anjos | Frente à Piscina | Subtidal 2	37.005815	-25.157587	Subtidal
9	SMA_VP_Apfem	Vila do Porto	Anjos | Ponta dos Frades | Entre-marés	37.012072	-25.146074	Intertidal
10	SMA_VP_apgrcn12s1	Vila do Porto	Área protegida de gestão de recursos da Costa Norte (SMA12) | Subtidal 1	37.01291	-25.14428	Subtidal
11	SMA_VP_apgrcn12s2	Vila do Porto	Área protegida de gestão de recursos da Costa Norte (SMA12) | Subtidal 2	37.02289	-25.08936	Subtidal
12	SMA_VP_apgrcs13s	Vila do Porto	Área protegida de gestão de recursos da Costa Sul (SMA13) | Subtidal	36.94455	-25.00806	Subtidal
13	SMA_VP_apgrpcpm21s1	Vila do Porto	Área Protegida de Gestão de Recursos da Ponta do Cintrão– Ponta da Maia (SMA21) | Subtidal 1	36.92892	-25.06439	Subtidal
14	SMA_VP_apgrpcpm21s2	Vila do Porto	Área Protegida de Gestão de Recursos da Ponta do Cintrão– Ponta da Maia (SMA21) | Subtidal 2	36.92489	-25.02421	Subtidal
15	SMA_VP_apgrpcpm21s3	Vila do Porto	Área Protegida de Gestão de Recursos da Ponta do Cintrão– Ponta da Maia (SMA21) | Subtidal 3	36.93505	-25.09226	Subtidal
16	SMA_VP_Apiem	Vila do Porto	Anjos | Piscina | Entre-marés	37.005173	-25.157061	Intertidal
17	SMA_VP_brsem	Vila do Porto	Boca da Ribeira Seca | Entre-marés	37.004435	-25.16595	Intertidal
18	SMA_VP_bss	Vila do Porto	Baixa do Sul | Subtidal	36.924751	-25.022099	Subtidal
19	SMA_VP_CBpes	Vila do Porto	Calheta de Baixo | Ponta das Eirinhas | Subtidal	36.933883	-25.014702	Subtidal
20	SMA_VP_crem	Vila do Porto	Calhau da Roupa | Entre-marés	36.9458	-25.146063	Intertidal
21	SMA_VP_Eem	Vila do Porto	Emissores | Entre-marés	36.998404	-25.175029	Intertidal
22	SMA_VP_FBbrs	Vila do Porto	Feteiras de Baixo | Baía do Raposo | Subtidal	37.010939	-25.118291	Subtidal
23	SMA_VP_Fem	Vila do Porto	Figueiral | Entre-marés	36.94574	-25.122836	Intertidal
24	SMA_VP_Fps	Vila do Porto	Figueiral | Ponta | Subtidal	36.94405	-25.122131	Subtidal
25	SMA_VP_ISLs	Vila do Porto	Ilhéu de São Lourenço | Subtidal	36.987488	-25.041122	Subtidal
26	SMA_VP_IVem	Vila do Porto	Ilhéu da Vila | Entre-marés	36.944045	-25.171163	Intertidal
27	SMA_VP_IVs1	Vila do Porto	Ilhéu da Vila | Subtidal 1	36.93948333	-25.17646667	Subtidal
28	SMA_VP_IVs10	Vila do Porto	Ilhéu da Vila | Subtidal 10	36.941005	-25.167868	Subtidal
29	SMA_VP_IVs2	Vila do Porto	Ilhéu da Vila | Subtidal 2	36.9388333	-25.1757	Subtidal
30	SMA_VP_IVs3	Vila do Porto	Ilhéu da Vila | Subtidal 3	36.9392	-25.17541667	Subtidal
31	SMA_VP_IVs4	Vila do Porto	Ilhéu da Vila | Subtidal 4	36.94125	-25.17528333	Subtidal
32	SMA_VP_IVs5	Vila do Porto	Ilhéu da Vila | Subtidal 5	36.939	-25.1752	Subtidal
33	SMA_VP_IVs6	Vila do Porto	Ilhéu da Vila | Subtidal 6	36.94318333	-25.17496667	Subtidal
34	SMA_VP_IVs7	Vila do Porto	Ilhéu da Vila | Subtidal 7	36.94045	-25.17448333	Subtidal
35	SMA_VP_IVs8	Vila do Porto	Ilhéu da Vila | Subtidal 8	36.9431	-25.17426667	Subtidal
36	SMA_VP_IVs9	Vila do Porto	Ilhéu da Vila | Subtidal 9	36.941125	-25.169649	Subtidal
37	SMA_VP_LApps	Vila do Porto	Lagoa | Pedra que Pica | Subtidal	36.931597	-25.075562	Subtidal
38	SMA_VP_Lbscs	Vila do Porto	Lagoinhas | Baía do Salto dos Cães | Subtidal	37.017358	-25.098105	Subtidal
39	SMA_VP_LIem	Vila do Porto	Lagoinhas | Entre-marés	37.015012	-25.085176	Intertidal
40	SMA_VP_LIfis	Vila do Porto	Lagoinhas | Fora do ilhéu | Subtidal	37.03565	-25.09881	Subtidal
41	SMA_VP_LIs	Vila do Porto	Lagoinhas | Subtidal	37.017954	-25.086356	Subtidal
42	SMA_VP_Mbcclnem	Vila do Porto	Maia | Baía entre Cedros e Castelete | lado Norte | Entre-marés	36.954591	-25.020362	Intertidal
43	SMA_VP_Mbcclsem	Vila do Porto	Maia | Baía entre Cedros e Castelete | lado Sul | Entre-marés	36.95264	-25.019663	Intertidal
44	SMA_VP_Mbcs	Vila do Porto	Maia | Baía dos Cedros | Subtidal	36.954952	-25.017313	Subtidal
45	SMA_VP_Mbs1	Vila do Porto	Maia | Baía | Subtidal 1	36.94436667	-25.00838333	Subtidal
46	SMA_VP_Mbs2	Vila do Porto	Maia | Baía | Subtidal 2	36.94393333	-25.00826667	Subtidal
47	SMA_VP_Mbs3	Vila do Porto	Maia | Baía | Subtidal 3	36.94433333	-25.00768333	Subtidal
48	SMA_VP_Mbs4	Vila do Porto	Maia | Baía | Subtidal 4	36.94235	-25.0076	Subtidal
49	SMA_VP_Mbs5	Vila do Porto	Maia | Baía | Subtidal 5	36.94318333	-25.00753333	Subtidal
50	SMA_VP_Mem	Vila do Porto	Maia | Entre-marés	36.943886	-25.014773	Intertidal
51	SMA_VP_Mfpis	Vila do Porto	Maia | Lado de Fora da Piscina | Subtidal	36.938923	-25.012707	Subtidal
52	SMA_VP_mfps1	Vila do Porto	Marina | Lado de fora do Pontão | Subtidal 1	36.944834	-25.146131	Subtidal
53	SMA_VP_mfps2	Vila do Porto	Marina | Lado de fora do Pontão | Subtidal 2	36.9458	-25.148333	Subtidal
54	SMA_VP_mpem	Vila do Porto	Marina | Pontão | Entre-marés	36.944396	-25.147067	Intertidal
55	SMA_VP_Mpiem	Vila do Porto	Maia | Piscina | Entre-marés	36.939526	-25.013879	Intertidal
56	SMA_VP_MPs1	Vila do Porto	Malbusca-Piedade | Subtidal 1	36.92783333	-25.0714	Subtidal
57	SMA_VP_MPs10	Vila do Porto	Malbusca-Piedade | Subtidal 10	36.929380	-25.071470	Subtidal
58	SMA_VP_MPs11	Vila do Porto	Malbusca-Piedade | Subtidal 11	36.930017	-25.071383	Subtidal
59	SMA_VP_MPs2	Vila do Porto	Malbusca-Piedade | Subtidal 2	36.92723333	-25.06591667	Subtidal
60	SMA_VP_MPs3	Vila do Porto	Malbusca-Piedade | Subtidal 3	36.9279	-25.07065	Subtidal
61	SMA_VP_MPs4	Vila do Porto	Malbusca-Piedade | Subtidal 4	36.927967	-25.072933	Subtidal
62	SMA_VP_MPs5	Vila do Porto	Malbusca-Piedade | Subtidal 5	36.92806667	-25.07045	Subtidal
63	SMA_VP_MPs6	Vila do Porto	Malbusca-Piedade | Subtidal 6	36.92621667	-25.06138333	Subtidal
64	SMA_VP_MPs7	Vila do Porto	Malbusca-Piedade | Subtidal 7	36.925667	-25.057567	Subtidal
65	SMA_VP_MPs8	Vila do Porto	Malbusca-Piedade | Subtidal 8	36.923030	-25.066550	Subtidal
66	SMA_VP_MPs9	Vila do Porto	Malbusca-Piedade | Subtidal 9	36.928750	-25.065217	Subtidal
67	SMA_VP_Ms1	Vila do Porto	Malbusca | Subtidal 1	36.93582965	-25.09382679	Subtidal
68	SMA_VP_Ms2	Vila do Porto	Malbusca | Subtidal 2	36.93821161	-25.07944033	Subtidal
69	SMA_VP_Ms3	Vila do Porto	Malbusca | Subtidal 3	36.938555	-25.085032	Subtidal
70	SMA_VP_PCbnss	Vila do Porto	Ponta do Castelo | Baía de Nossa Senhora | Subtidal	36.931039	-25.057255	Subtidal
71	SMA_VP_PCem	Vila do Porto	Ponta do Castelo | Entre-marés	36.928153	-25.017055	Intertidal
72	SMA_VP_PCras	Vila do Porto	Ponta do Castelo | Rocha Alta | Subtidal	36.926463	-25.014565	Subtidal
73	SMA_VP_Pem	Vila do Porto	Prainha | Entre-marés	36.951808	-25.104061	Intertidal
74	SMA_VP_PFepem	Vila do Porto	Praia Formosa | Entre praias | Entre-marés	36.950235	-25.095009	Intertidal
75	SMA_VP_PFppem	Vila do Porto	Praia Formosa | Ponta da praia | Entre-marés	36.94734	-25.088821	Intertidal
76	SMA_VP_PFps	Vila do Porto	Praia Formosa | Pedrinha | Subtidal	36.937365	-25.105259	Subtidal
77	SMA_VP_PFs1	Vila do Porto	Praia Formosa | Subtidal 1	36.940431	-25.095659	Subtidal
78	SMA_VP_PMs	Vila do Porto	Ponta do Marvão | Subtidal	36.936973	-25.139363	Subtidal
79	SMA_VP_Rs	Vila do Porto	Restinga | Subtidal	37.001733	-25.172973	Subtidal
80	SMA_VP_SLaps	Vila do Porto	São Lourenço | Atrás do porto | Subtidal	36.99533	-25.052727	Subtidal
81	SMA_VP_SLb11s	Vila do Porto	São Lourenço | Baía (SMA11) | Subtidal	36.98472	-25.04341	Subtidal
82	SMA_VP_SLfiem	Vila do Porto	São Lourenço | Frente ao ilhéu | Entre-marés	36.9858	-25.049216	Intertidal
83	SMA_VP_Slpnem	Vila do Porto	São Lourenço | Ponta Norte | Entre-marés	36.998556	-25.050887	Intertidal
84	SMA_VP_SLpns	Vila do Porto	São Lourenço | Ponta do Norte | Subtidal	37.00491	-25.05133	Subtidal
85	SMA_VP_Slpsbem	Vila do Porto	São Lourenço | Ponta Sul da Baía | Entre-marés	36.98538307	-25.05051544	Intertidal
86	SMA_VP_SLs1	Vila do Porto	São Lourenço | Subtidal 1	36.996286	-25.045811	Subtidal
87	SMA_VP_SLs2	Vila do Porto	São Lourenço | Subtidal 2	36.997331	-25.047914	Subtidal
88	SMA_VP_VPpaem	Vila do Porto	Vila do Porto | Porto antigo | Entre-marés	36.945957	-25.14822	Intertidal
89	SMA_VP_VPpnemW	Vila do Porto	Vila do Porto | Porto Novo | Entre-marés W	36.94141	-25.154005	Intertidal
90	SMA_VP_VPpns	Vila do Porto	Vila do Porto | Porto Novo | Subtidal	36.940838	-25.146736	Subtidal
91	SMA_VP_VPpnsE	Vila do Porto	Vila do Porto | Porto Novo | Subtidal E	36.9431	-25.146917	Subtidal
92	SMA_VP_VPpnsW	Vila do Porto	Vila do Porto | Porto Novo | Subtidal W	36.9402	-25.150384	Subtidal

**Table 3.** Macroalgae species recorded from Santa Maria Island, with information on relative abundance, origin and status

**Phylum**	**Species (Accepted Name)**	**Number of records**	**Establishment Means**	**OccurrenceRemarks**
Rhodophyta	*Acrosorium ciliolatum* (Harvey) Kylin	6	Native	
Rhodophyta	*Acrothamnion preissii* (Sonder) E.M.Wollaston	28	Introduced	
Rhodophyta	*Aglaothamnion pseudobyssoides* (Crouan & Crouan) Halos	1	Native	New record
Rhodophyta	*Amphiroa fragilissima* (Linnaeus) J.V.Lamouroux	1	Native	New record
Rhodophyta	*Anotrichium furcellatum* (J.Agardh) Baldock	6	Uncertain	
Rhodophyta	*Antithamnion hubbsii* E.Y.Dawson	5	Introduced	New record
Rhodophyta	*Asparagopsis armata* Harvey	22	Introduced	
Rhodophyta	*Asparagopsis armata* Harvey phase *Falkenbergia rufolanosa* (Harvey) F.Schmitz	16	Introduced	
Rhodophyta	*Asparagopsis taxiformis* (Delile) Trevisan	66	Native	
Rhodophyta	*Bonnemaisonia hamifera* Hariot	3	Introduced	New record
Rhodophyta	*Bornetia secundiflora* (J.Agardh) Thuret	1	Native	New record
Rhodophyta	*Callithamnion corymbosum* (J.E.Smith) Lyngbye	2	Native	
Rhodophyta	*Callithamnion granulatum* (Ducluzeau) C.Agardh	4	Native	
Rhodophyta	*Carradoriella denudata* (Dillwyn) A.M.Savoie & G.W.Saunders	3	Uncertain	
Rhodophyta	*Carradoriella elongata* (Hudson) A.M.Savoie & G.W.Saunders	5	Native	
Rhodophyta	*Catenella caespitosa* (Withering) L.M.Irvine	3	Native	
Rhodophyta	*Caulacanthus ustulatus* (Turner) Kützing	6	Uncertain	
Rhodophyta	*Centroceras clavulatum* (C.Agardh) Montagne	8	Native	
Rhodophyta	*Ceramium codii* (H.Richards) Mazoyer	1	Native	New record
Rhodophyta	*Ceramium diaphanum* (Lightfoot) Roth	10	Native	
Rhodophyta	*Ceramium strictum* Roth	1	Native	
Rhodophyta	*Ceramium virgatum* Roth	5	Native	
Rhodophyta	*Chondracanthus acicularis* (Roth) Fredericq	6	Native	
Rhodophyta	*Chondracanthus teedei* (Mertens ex Roth) Kützing	3	Native	
Rhodophyta	*Chondria capillaris* (Hudson) M.J.Wynne	2	Native	
Rhodophyta	*Chondria dasyphylla* (Woodward) C.Agardh	19	Uncertain	
Rhodophyta	*Corallina ferreyrae* E.Y.Dawson, Acleto & Foldvik	3	Native	New record
Rhodophyta	*Corallina officinalis* Linnaeus	5	Native	
Rhodophyta	*Cottoniella filamentosa* (M.Howe) Børgesen	30	Native	New record
Rhodophyta	*Crouania attenuata* (C.Agardh) J.Agardh	1	Native	New record
Rhodophyta	*Cryptopleura ramosa* (Hudson) L.Newton	19	Native	
Rhodophyta	*Dasya baillouviana* (S.G.Gmelin) Montagne	3	Uncertain	New record
Rhodophyta	*Dasya corymbifera* J.Agardh	3	Native	
Rhodophyta	*Dasya hutchinsiae* Harvey	2	Native	
Rhodophyta	*Dasya rigidula* (Kützing) Ardissone	2	Native	New record
Rhodophyta	*Dermocorynus dichotomus* (J.Agardh) Gargiulo, M.Morabito & Manghisi	1	Native	
Rhodophyta	*Dudresnaya verticillata* (Withering) Le Jolis	1	Native	
Rhodophyta	*Ellisolandia elongata* (J.Ellis & Solander) K.R.Hind & G.W.Saunders	6	Native	
Rhodophyta	*Erythrocystis montagnei* (Derbès & Solier) P.C.Silva	2	Native	
Rhodophyta	*Feldmannophycus rayssiae* (Feldmann & G.Feldmann) H.Augier & Boudouresque	1	Native	New record
Rhodophyta	*Gaillona hookeri* (Dillwyn) Athanasiadis	6	Native	
Rhodophyta	*Gelidium corneum* (Hudson) J.V.Lamouroux	3	Native	New record
Rhodophyta	*Gelidium microdon* Kützing	11	Native	
Rhodophyta	*Gelidium pusillum* (Stackhouse) Le Jolis	1	Native	
Rhodophyta	*Gelidium spinosum* (S.G.Gmelin) P.C.Silva	2	Native	
Rhodophyta	*Gigartina pistillata* (S.G.Gmel.) Stackhouse	3	Native	
Rhodophyta	*Gracilariopsis longissima* (S.G.Gmelin) Steentoft, L.M.Irvine & Farnham	14	Native	
Rhodophyta	*Grateloupia filicina* (J.V.Lamouroux) C.Agardh	16	Native	
Rhodophyta	*Griffithsia corallinoides* (Linnaeus) Trevisan	1	Uncertain	
Rhodophyta	*Gymnogongrus crenulatus* (Turner) J.Agardh	3	Native	
Rhodophyta	*Gymnogongrus griffithsiae* (Turner) C.Martius	4	Native	
Rhodophyta	*Halarachnion ligulatum* (Woodward) Kützing	1	Native	New record
Rhodophyta	*Halurus equisetifolius* (Lightfoot) Kützing	1	Native	New record
Rhodophyta	*Halurus flosculosus* (J.Ellis) Maggs & Hommersand	6	Native	
Rhodophyta	*Herposiphonia secunda* (C.Agardh) Ambronn	2	Native	
Rhodophyta	Herposiphonia secunda f. tenella (C.Agardh) M.J.Wynne	2	Native	New record
Rhodophyta	*Hypnea musciformis* (Wulfen) J.V.Lamouroux	21	Uncertain	
Rhodophyta	*Hypoglossum hypoglossoides* (Stackhouse) F.S.Collins & Hervey	1	Native	
Rhodophyta	*Itonoa marginifera* (J.Agardh) Masuda & Guiry	1	Native	New record
Rhodophyta	*Jania capillacea* Harvey	1	Native	
Rhodophyta	*Jania longifurca* Zanardini	2	Uncertain	
Rhodophyta	Jania pedunculata var. adhaerens (J.V.Lamouroux) A.S.Harvey, Woelkerling & Reviers	5	Native	New record
Rhodophyta	*Jania rubens* (Linnaeus) J.V.Lamouroux	11	Native	
Rhodophyta	*Jania virgata* (Zanardini) Montagne	25	Uncertain	
Rhodophyta	*Laurencia obtusa* (Hudson) J.V.Lamouroux	2	Native	
Rhodophyta	*Laurencia pyramidalis* Bory ex Kützing	4	Native	New record
Rhodophyta	*Laurencia tenera* C.K.Tseng	1	Native	New record
Rhodophyta	*Laurencia viridis* Gil-Rodriguez & Haroun	111	Macaronesian endemism	
Rhodophyta	*Leptosiphonia brodiei* (Dillwyn) A.M.Savoie & G.W.Saunders	3	Uncertain	
Rhodophyta	*Liagora distenta* (Mertens ex Roth) J.V.Lamouroux	4	Native	New record
Rhodophyta	*Liagora viscida* (Forsskål) C.A.Agardh	6	Native	New record
Rhodophyta	*Lophosiphonia cristata* Falkenberg	2	Native	
Rhodophyta	*Melanothamnus harveyi* (Bailey) Díaz-Tapia & Maggs	2	Introduced	New record
Rhodophyta	*Meredithia microphylla* (J.Agardh) J.Agardh	11	Native	
Rhodophyta	*Millerella tinerfensis* (Seoane-Camba) S.M.Boo & J.M.Rico	1	Macaronesian endemism	
Rhodophyta	*Nemalion elminthoides* (Velley) Batters	4	Native	
Rhodophyta	*Nitophyllum punctatum* (Stackhouse) Greville	2	Native	
Rhodophyta	*Osmundea pinnatifida* (Hudson) Stackhouse	7	Native	
Rhodophyta	*Osmundea truncata* (Kützing) K.W.Nam & Maggs	1	Native	
Rhodophyta	*Peyssonnelia squamaria* (S.G.Gmelin) Decaisne ex J.Agardh	1	Native	
Rhodophyta	*Phyllophora crispa* (Hudson) P.S.Dixon	6	Native	New record
Rhodophyta	*Platoma cyclocolpum* (Montagne) F.Schmitz	8	Native	
Rhodophyta	*Platysiphonia delicata* (Clemente) Cremades	2	Native	New record
Rhodophyta	*Pleonosporium borreri* (Smith) Nägeli	7	Native	New record
Rhodophyta	*Plocamium cartilagineum* (Linnaeus) P.S.Dixon	22	Native	
Rhodophyta	*Polysiphonia atlantica* Kapraun & J.N.Norris	2	Native	
Rhodophyta	*Polysiphonia breviarticulata* (C.Agardh) Zanardini	1	Native	New record
Rhodophyta	*Polysiphonia ceramiiformis* P.Crouan & H.Crouan	1	Native	
Rhodophyta	*Polysiphonia havanensis* Montagne	2	Native	
Rhodophyta	*Predaea feldmannii* Børgesen	9	Native	New record
Rhodophyta	*Pterocladiella capillacea* (S.G.Gmelin) Santelices & Hommersand	41	Native	
Rhodophyta	*Rhodymenia holmesii* Ardissone	6	Native	
Rhodophyta	*Scinaia acuta* M.J.Wynne	2	Introduced	
Rhodophyta	*Scinaia furcellata* (Turner) J.Agardh	2	Native	
Rhodophyta	*Sphaerococcus coronopifolius* Stackhouse	13	Native	New record
Rhodophyta	*Sphondylothamnion multifidum* (Hudson) Nägeli	1	Native	
Rhodophyta	*Spyridia filamentosa* (Wulfen) Harvey	8	Native	
Rhodophyta	*Symphyocladia marchantioides* (Harvey) Falkenberg	5	Introduced	
Rhodophyta	*Taenioma nanum* (Kützing) Papenfuss	1	Native	
Rhodophyta	*Vertebrata foetidissima* (Cocks ex Bornet) Díaz-Tapia & Maggs	1	Native	New record
Rhodophyta	*Vertebrata fruticulosa* (Wulfen) Kuntze	9	Native	
Rhodophyta	*Vertebrata fucoides* (Hudson) Kuntze	3	Uncertain	
Rhodophyta	*Xiphosiphonia pennata* (C.Agardh) Savoie & G.W.Saunders	5	Native	
Chlorophyta	*Bryopsis hypnoides* J.V.Lamouroux	3	Native	
Chlorophyta	*Bryopsis plumosa* (Hudson) C.Agardh	1	Native	
Chlorophyta	*Chaetomorpha aerea* (Dillwyn) Kützing	3	Native	
Chlorophyta	*Chaetomorpha linum* (O.F.Müller) Kützing	7	Native	
Chlorophyta	*Chaetomorpha pachynema* (Montagne) Kützing	1	Native	
Chlorophyta	*Cladophora albida* (Nees) Kützing	6	Native	
Chlorophyta	*Cladophora coelothrix* Kützing	6	Native	
Chlorophyta	*Cladophora laetevirens* (Dillwyn) Kützing	10	Uncertain	
Chlorophyta	*Cladophora lehmanniana* (Lindenberg) Kützing	4	Native	New record
Chlorophyta	*Cladophora liebetruthii* Grunow	9	Native	
Chlorophyta	*Cladophora prolifera* (Roth) Kützing	42	Native	
Chlorophyta	*Codium adhaerens* C.Agardh	43	Native	
Chlorophyta	*Codium effusum* (Rafinesque) Delle Chiaje	1	Uncertain	New record
Chlorophyta	Codium fragile subsp. atlanticum (A.D.Cotton) P.C.Silva	1	Native	New record
Chlorophyta	Codium fragile subsp. fragile (Suringar) Hariot	13	Introduced	New record
Chlorophyta	*Codium taylorii* P.C.Silva	4	Native	New record
Chlorophyta	*Codium tomentosum* Stackhouse	1	Native	
Chlorophyta	*Lychaete pellucida* (Hudson) M.J.Wynne	5	Native	
Chlorophyta	*Microdictyon umbilicatum* (Velley) Zanardini	8	Native	New record
Chlorophyta	*Pseudorhizoclonium africanum* (Kützing) Boedeker	1	Native	New record
Chlorophyta	*Ulothrix flacca* (Dillwyn) Thuret	1	Native	New record
Chlorophyta	*Ulva clathrata* (Roth) C.Agardh	13	Native	
Chlorophyta	*Ulva compressa* Linnaeus	12	Native	
Chlorophyta	*Ulva intestinalis* Linnaeus	13	Native	
Chlorophyta	*Ulva lactuca* Linnaeus	3	Uncertain	New record
Chlorophyta	*Ulva linza* Linnaeus	2	Native	
Chlorophyta	*Ulva rigida* C.Agardh	25	Native	
Chlorophyta	*Valonia macrophysa* Kützing	1	Native	
Chlorophyta	*Valonia utricularis* (Roth) C.Agardh	7	Native	
Ochrophyta	*Bachelotia antillarum* (Grunow) Gerloff	*1*	Native	
Ochrophyta	*Canistrocarpus cervicornis* (Kützing) De Paula & De Clerck	*1*	Native	New record
Ochrophyta	*Carpomitra costata* (Stackhouse) Batters	2	Native	New record
Ochrophyta	*Cladostephus spongiosus* (Hudson) C.Agardh	44	Native	
Ochrophyta	*Colpomenia sinuosa* (Mertens ex Roth) Derbès & Solier	90	Native	
Ochrophyta	*Cutleria multifida* (Turner) Greville	2	Uncertain	New record
Ochrophyta	*Cutleria multifida* (Turner) Greville phase *Aglaozonia parvula* (Greville) Zanardini	2	Uncertain	
Ochrophyta	*Cystoseira compressa* (Esper) Gerloff & Nizamuddin	17	Native	New record
Ochrophyta	*Cystoseira foeniculacea* (Linnaeus) Greville	2	Native	
Ochrophyta	*Cystoseira humilis* Schousboe ex Kützing	7	Native	
Ochrophyta	*Cystoseira tamariscifolia* (Hudson) Papenfuss	5	Native	
Ochrophyta	*Dictyopteris polypodioides* (A.P.De Candolle) J.V.Lamouroux	8	Native	
Ochrophyta	*Dictyota bartayresiana* J.V.Lamouroux	3	Native	
Ochrophyta	*Dictyota ciliolata* Sonder ex Kützing	1	Native	
Ochrophyta	*Dictyota dichotoma* (Hudson) J.V.Lamouroux	24	Native	
Ochrophyta	Dictyota dichotoma var. intricata (C.Agardh) Greville	11	Native	New record
Ochrophyta	*Dictyota implexa* (Desfontaines) J.V.Lamouroux	2	Native	
Ochrophyta	*Feldmannia globifera* (Kützing) Hamel	1	Native	New record
Ochrophyta	*Fucus spiralis* Linnaeus	27	Uncertain	
Ochrophyta	*Halopteris filicina* (Grateloup) Kützing	37	Native	
Ochrophyta	*Halopteris scoparia* (Linnaeus) Sauvageau	54	Native	
Ochrophyta	*Hydroclathrus tilesii* (Endlicher) Santiañez & M.J.Wynne	8	Introduced	New record
Ochrophyta	*Hydroclathrus clathratus* (C.Agardh) M.Howe	6	Native	
Ochrophyta	*Leathesia marina* (Lyngbye) Decaisne	9	Uncertain	
Ochrophyta	*Lobophora variegata* (J.V.Lamouroux) Womersley ex E.C.Oliveira	41	Native	
Ochrophyta	*Mesogloia vermiculata* (Smith) S.F.Gray	16	Native	New record
Ochrophyta	*Myrionema strangulans* Greville	8	Native	
Ochrophyta	*Nemoderma tingitanum* Schousboe ex Bornet	3	Native	
Ochrophyta	*Padina pavonica* (Linnaeus) Thivy	144	Native	
Ochrophyta	*Papenfussiella kuromo* (Yendo) Inagaki	8	Introduced	
Ochrophyta	*Ralfsia verrucosa* (Areschoug) Areschoug	1	Native	New record
Ochrophyta	*Sargassum cymosum* C.Agardh	8	Native	
Ochrophyta	*Sargassum desfontainesii* (Turner) C.Agardh	3	Native	
Ochrophyta	*Sargassum furcatum* Kützing	16	Native	New record
Ochrophyta	*Sargassum vulgare* C.Agardh, nom. illeg.	2	Native	
Ochrophyta	*Scytosiphon lomentaria* (Lyngbye) Link	5	Native	
Ochrophyta	*Sphacelaria cirrosa* (Roth) C.Agardh	6	Native	
Ochrophyta	*Sphacelaria plumula* Zanardini	2	Native	
Ochrophyta	*Sphaerotrichia divaricata* (C.Agardh) Kylin	4	Uncertain	New record
Ochrophyta	*Sporochnus pedunculatus* (Hudson) C.Agardh	2	Native	New record
Ochrophyta	*Stypopodium zonale* (J.V.Lamouroux) Papenfuss	1	Native	New record
Ochrophyta	*Taonia atomaria* (Woodward) J.Agardh	3	Native	
Ochrophyta	*Treptacantha abies-marina* (S.G.Gmelin) Kützing	35	Native	
Ochrophyta	*Zonaria tournefortii* (J.V.Lamouroux) Montagne	100	Native	

**Table 4.** Summary of the macroalgal flora of the Island of Santa Maria with information on the species origin and status

Phyllum	Order	Family	Specimens Number	Total taxa	Total species	Native	Introduced	Uncertain	Macaronesian endemism	New record
Rhodophyta	14	34	988	152	102	82	7	11	2	30
Chlorophyta	5	9	276	43	29	25	1	3		9
Ochrophyta	9	17	1065	66	44	37	2	4		13
Total	28	60	2329	261	174	144	10	18	2	52

## References

[B6373543] Afonso-Carrillo J., Sansón M. (1989). Clave ilustrada para la determinación de los macrófitos marinos bentónicos de las Islas Canarias.

[B6373608] Agardh J. G. (1870). Om de under Korvetten Josephines expedition, sistliden sommar, insamlade Algerne, ofversigt of Kongl. Vetenskaps-Akademiens Forhanlingar, Stockholm.

[B6373617] Amen R. G., Neto A. I., Azevedo J. M.N. (2005). Coralline-algal framework in the Quaternary of Prainha (Santa Maria Island, Azores. Revista Espaola de Micropaleontologa.

[B6373635] Ardré F., Boudouresque C. -F., Cabioch J. (1974). *Symphyocladia
marchantioides* (Harvey) Falkenberg (Rhodomeniaceae, Ceramiales) aux Aores. Bulletin de la Societe Phycologique de France.

[B6373646] Ávila S. P., Ramalho R. S., Habermann J. M., Quartau R., Kroh A., Berning B., Johnson M., Kirby M. X., Zanon V., Titschack J., Goss A., Rebelo A. C., Melo C., Madeira P., Cordeiro R., Meireles R., Bagaço L., Hiplito A., Uchman A., Silva C. M., Cachão M., Madeira J. (2015). Palaeoecology, taphonomy, and preservation of a lower Pliocene shell bed (coquina) from a volcanic oceanic island (Santa Maria Island, Azores). Palaeogeography, Palaeoclimatology, Palaeoecology.

[B6373673] Ávila S. P., Cachão M., Ramalho R. S., Botelho A. Z., Madeira P., Rebelo A. C., Cordeiro R., Melo C., Hipólito A., Ventura M., Lipps J. H. (2016). The palaeontological heritage of Santa Maria Island (Azores: NE Atlantic): a re-evaluation of geosites in GeoPark Azores and their use in geotourism. Geoheritage.

[B6373754] Azevedo J. M. N., Álvaro N. V., Raposeiro P., Neto A. I. (2008). Guias Costeiros de Santa Maria: Peixes Litorais.

[B6373787] Botelho A. Z., Dionísio M. A., Cunha A., Torres P., Monteiro S., Geraldes D., Costa A. C. (2010). Contributo para a inventariação da biodiversidade marinha da ilha de Santa Maria.

[B6373835] Boudouresque C. - F., Meinesz A., Verlaque M., Boudouresque C. - F. (1992). Méditerranée. Guide des Algues des Mers d'Europe.

[B6373873] Bridsen D., Forman L. (1999). The Herbarium Handbook. Third Edition.

[B6373907] Brodie J., Maggs C., John D. M. (2007). The green seaweeds of Britain and Ireland.

[B6373926] Burrows E. M. (1991). Seaweeds of the British lsles. Vol. 2. Chlorophyta.

[B6373944] Cabioc'h J., Floc'h J. Y., Le Toquin A., Boudouresque C. - F. (1992). Manche et Atlantique. Guide des Algues des Mers d'Europe.

[B6373966] Cardoso P., Erwin T., Borges P. V., New T. (2011). The seven impediments in invertebrate conservation and how to overcome them. Biological Conservation.

[B6373984] Dixon S. P., Irvine L. M. (1977). Seaweeds of the British Isles. Vol. I Rhodophyta. Part 1 Introduction, Nemaliales, Gigartinales.

[B6374032] Drouët H. (1866). Catalogue de la flore des lles Açores précédé de l'itinéraire d'un voyage dans cet Archipel. Memoires de la Société Académique de l'Aube.

[B6374118] Fletcher R. L. (1987). Seaweeds of the British Isles. Vol. III. Fucophyceae (Phaeophyceae). Part 1.

[B6374126] Fralick R. A., Hehre E. J. (1990). Observations on the marine algal flora of the Azores II. An annotated checklist of the Chlorophyta of the Azores. Arquipelago (Life and Earth Sciences).

[B6374135] Freitas R., Romeiras M., Silva L., Cordeiro R., Madeira P., González J. A., Wirtz P., Falcón J. M., Brito A., Floeter S. R., Afonso P., Porteiro F., Viera-Rodríguez M. A., Neto A. I., Haroun R., Farminho J. N.M., Rebelo A. C., Baptista L., Melo C. S., Martínez A., Núñez J., Berning B., Johnson M. E., Ávila S. P. (2019). Restructuring of the Macaronesia biogeographic unit: A marine multi-taxon biogeographical approach. Scientific Reports.

[B6374164] Gayral P., Cosson J. (1986). Connaitre et reconnaitre les algues marines.

[B6374172] Guiry M. D., Guiry G. M. AlgaeBase. World-wide electronic publication, National University of Ireland, Galway. https://www.algaebase.org.

[B6374200] Hidrográfico I. (1981). Roteiro do Arquipélago dos Açores. PUB. (N) -lli-128-SN, Lisboa.

[B6374180] Hildenbrand A., Weis D., Madureira P., Marques F. O. (2014). Recent plate re-organization at the Azores Triple Junction: Evidence from combined geochemical and geochronological data on Faial, S. Jorge and Terceira volcanic Islands. Lithos.

[B6374189] Hortal J., Bello F., Diniz-Filho J. A.F., Lewinsohn T. M., Lobo J. M., Ladle R. J. (2015). Seven shortfalls that beset large-scale knowledge of biodiversity. Annual Review of Ecology, Evolution, and Systematics.

[B6374208] Irvine L. M. (1983). Seaweeds of the British Isles. Vol. I Rhodophyta. Part 2 A Cryptonemiales (sensu stricto), Palmariales, Rhodymeniales.

[B6374216] Irvine L. M., Chamberlain Y. M. (1994). Seaweeds of the British Isles. Vol. 1. Rhodophyta. Part 2B. Corallinales, Hildenbrandiales.

[B6374224] Johnson M. E., Ledesma-Vzquez J., Ramalho R. S., Silva C. M., Rebelo A. C., Santos A., Baarli B. G., Mayoral E., Cachão M., Riosmena-Rodrguez R., Nelson W., Aguirre J. (2017). Chapter 9. Taphonomic Range and Sedimentary Dynamics of Modern and Fossil Rhodolith Beds: Macaronesian Realm (North Atlantic Ocean). Rhodolith/ Maërl Beds: A global Perspective.

[B6374242] Lawson G. W., John D. M. (1982). The marine algae and coastal environment of Tropical West Africa.

[B6374251] León-Cisneros K., Riosmena-Rodríguez R., Neto A. I. (2011). A re-evaluation of *Scinaia* (Nemaliales, Rhodophyta) in the Azores. Helgolander Marine Research.

[B6374260] Levring T. (1974). The marine algae of the archipelago of Madeira. Boletim do Museu Municipal do Funchal.

[B6374269] Lloréns J. L. P., Cabrero I. H., Lacida R. B., González G. P., Murillo F. G. B., Oñate J. J. V. (2012). Flora marina del litoral gaditano. Biología, ecología, usos y guía de identificación. 368 pp. mCN Monografias de Ciencias de la Naturaleza. Servicio de Publicaciones de la Universidad de Cádiz, Cádiz.

[B6374279] Machín-Sánchez M., Rousseau F., Le Gall L., Cassano V., Neto A. I., Sentíes A., Fujii M. T., Gil-Rodrguez M. C. (2016). Species diversity of the genus *Osmundea* (Ceramiales, Rhodophyta) in the Macaronesian region. Journal of Phycology.

[B6374292] Maggs C. A., Hommersand M. H. (1993). Seaweeds of the British Isles. Vol1. Rhodophyta. Part 3A. Ceramiales.

[B6374300] Martins G. M., Faria J., Furtado M., Neto A. I. (2014). Shells of Patella
aspera as islands for epibionts. Journal of the Marine Biological Association of the United Kingdom.

[B6374309] Micael J., Parente M. I., Costa A. C. (2014). Tracking macroalgae introductions in North Atlantic oceanic islands. Helgoland Marine Research.

[B6374327] Morton B., Britton J. C., Martins A. M. F. (1998). Coastal Ecology of the Azores.

[B6374318] Morton B., Britton J. C. (2000). Origins of the Azorean intertidal biota: the significance of introduced species, survivors of chance events. Arquipelago Life Marine Science Suppl.

[B6374335] Neto A. I., Baldwin H. P., Fralick R. A., Hehre E. J. (1991). Algas marinhas do litoral da ilha de Santa Maria. Santa Maria e Formigas/90, Relatório Preliminar. Relatórios e Comunicações do Departamento de Biologia, 19: 27-32.

[B6374343] Neto A. I., Tittley I., Raposeiro P. (2005). Flora Marinha do Litoral dos Açores.

[B6374369] Neto A. I., Wallenstein F. M., Álvaro N. V., N. Azevedo J. M. (2008). Guias Costeiros de Santa Maria: Zona Submersa.

[B6374351] Neto A. I., Wallenstein F. M., Silva T. P., Álvaro N. V., Tittley I. (2008). Guias Costeiros de Santa Maria: Poças de Maré.

[B6374360] Neto A. I., Wallenstein F. M., Silva T. P., Álvaro N. V., Tittley I. (2008). Guias Costeiros de Santa Maria: Zona Entre-Marés.

[B6374378] Neto A. I., Prestes A. C.L., Álvaro N. V., Resendes R., Neto R. M.A., Moreu I. (2020). Marine algal (seaweed) flora of Terceira Island, Azores. Biodiversity Data Journal.

[B6374389] Neto A. I., Prestes A. C.L., Álvaro N. V., Resendes R., Neto R. M.A., Tittley I., Moreu I. (2020). Marine algal flora of Pico Island, Azores. Biodiversity Data Journal.

[B6374401] Neto A. I., Parente M. I., Botelho A. Z., Prestes A. C.L., Resendes R., Afonso C. L.P., Álvaro N. V., Milla-Figueras D., Neto R. M.A., Tittley I., Moreu I. (2020). Marine algal flora of Graciosa Island, Azores. Biodiversity Data Journal.

[B6374617] Neto A. I., Parente M. I., Cacabelos E., Costa A. C., Botelho A. Z., Ballesteros E., Monteiro S., Resendes R., Afonso P., Afonso C. L.P., Patarra R. F., Álvaro N. V., Milla-Figueras D., Neto R. M. A., Azevedo J. M. N., Moreu I. (2020). Marine algal flora of Santa Maria Island, Azores. Version 1.2. Universidade dos Açores. Sampling event dataset.

[B6374417] Neto A. I., Parente M. I., Tittley I., Fletcher R. L., Farnham W. F., Costa A. C., Botelho A. Z., Monteiro S., Resendes R., Afonso P., Prestes A. C. L., Álvaro N. V., Milla-Figueras D., Neto R. M. A., Azevedo J. M. N., Moreu I. (2020). Marine algal flora of Flores and Corvo Islands, Azores. v1.4. Sampling event dataset.

[B6374438] Parente M. I., Gabriel D., Micael J., Botelho A. Z., Ballesteros E., Milla D., Santos R., Costa A. C. (2018). First report of the invasive macroalga *Acrothamnion
preissii* (Rhodophyta, Ceramiales) in the Atlantic Ocean. Botanica Marina.

[B6374451] Rebelo A. C., Rasser M. W., Riosmena-Rodriguez R., Neto A. I., Ávila S. P. (2014). Rhodolith forming coralline algae in the Upper Miocene of Santa Maria Island (Azores, NE Atlantic): a critical evaluation. Phytotaxa.

[B6374461] Rodríguez-Prieto C., Ballesteros E., Boisset F., Afonso-Carrillo J. (2013). Guía de las macroalgas y fanerógamas marinas del Mediterráneo Occidental.

[B6374469] Schmidt O. C. (1931). Die marine vegetation der Azoren in ihren Grundzgen dargestellt. Bibliotheca Botanica.

[B6374478] Taylor W. R. (1967). Marine algae of the northeastern coasts of North America.

[B6374509] Taylor W. R. (1978). Marine algae of the eastern tropical and subtropical coasts of the Americas.

[B6374517] Tittley I. (2003). Seaweed diversity in the North Atlantic Ocean. Arquipelago. Life and Marine Sciences.

[B6374526] Tittley I., Neto A. I. (2005). The marine algal (seaweed) flora of the Azores: additions and amendments. Botanica Marina.

[B6374535] Tittley I., Neto A. I. (2006). The marine algal flora of the Azores: Island isolation or Atlantic stepping stones?. Occasional papers of the Irish Biogeographical Society.

[B6374544] Tittley I., Neto A. I., Parente M. I. (2009). The marine algal (seaweed) flora of the Azores: additions and amendments 3. Botanica Marina.

[B6374553] Torres P., Lopes C., Dionísio M. A., Costa A. C. (2010). Espécies exóticas invasoras marinhas da ilha de Santa Maria, Açores. XIV Expedição Científica do Departamento de Biologia - Santa Maria 2009..

[B6374561] Trelease W. (1897). Botanical observations on the Azores. 8th Annual Report of the Michigan Botanical Garden: 77-220.

[B6374569] Uchman A., Johnson M. E., Rebelo A. C., Melo A. C., Cordeiro R., Ramalho R. S., Ávila S. P. (2016). Vertically-oriented trace fossil Macaronichnus segregatis from Neogene of Santa Maria Island (Azores; NE Atlantic) records vertical fluctuations of the coastal groundwater mixing zone on a small oceanic island. Geobios.

[B6374581] Wallenstein F. M., Neto A. I. (2006). Intertidal rocky shore biotopes of the Azores: a quantitative approach. Helgoland Marine Research.

[B6374590] Wallenstein F. M., Terra M. R., Pombo J., Neto A. I. (2009). Macroalgal turfs in the Azores. Marine Ecology - An Evolutionary Perspective.

[B6374599] Wallenstein F. M., Neto A. I., Álvaro N. V., Tittley I., Azevedo J. M. N (2009). Guia para Definição de Biótopos Costeiros em Ilhas Oceânicas.

[B6374608] Wallenstein F. M., Peres S. D., Xavier E. D., Neto A. I. (2010). Phytobenthic communities of intertidal rock pools in the eastern islands of Azores and their relation to position on shore and pool morphology. Arquipélago. Life and Marine Sciences.

